# Aging of Tire Particles in Deep-Sea Conditions: Interactions between Hydrostatic Pressure, Prokaryotic Growth and Chemical Leaching

**DOI:** 10.1021/acs.est.5c10705

**Published:** 2025-09-17

**Authors:** Natascha Schmidt, Aurelio Foscari, Dorte Herzke, Marc Garel, Christian Tamburini, Bettina Seiwert, Thorsten Reemtsma, Richard Sempéré

**Affiliations:** † 87482 NILU, The FRAM Centre, P.O. Box 6606, Tromsø 9296, Norway; ‡ Aix-Marseille Univ., Université de Toulon, CNRS, IRD, MIO, Marseille 13009, France; § Helmholtz Centre for Environmental Research—UFZ, Department of Environmental Analytical Chemistry, Leipzig 04318, Germany; ∥ Institute for Analytical Chemistry, University of Leipzig, Leipzig 04103, Germany; ⊥ Aix-Marseille Univ., CNRS, LCE, UM 7376, Ocean Sciences Institute, Marseille 13003, France

**Keywords:** additives, deep-sea marine pollution, weathered rubber particles, CMTT, microplastics, high-hydrostatic pressure, prokaryotes

## Abstract

Tire particles can enter the marine environment e.g. through direct discharge of road runoff, sewage systems or riverine inputs. Their fate in marine waters remains largely unknown, though the deep sea could be a final sink as for other marine litter. To simulate these conditions, we investigated in laboratory-controlled conditions the effects of high-hydrostatic pressure [20 MPa] vs atmospheric pressure [0.1 MPa] on the leaching of 17 organic compounds from cryo-milled tire tread particles (μm sized) and crumb rubber particles (mm sized) into natural seawater. We monitored the abundance of heterotrophic prokaryotes in the leachates over the 14 day exposure period under biotic conditions. Abiotic controls were employed to delineate the influence of prokaryotes on the fate of leached chemicals. Our results showed leaching of dissolved organic carbon and target chemicals under all experimental conditions, with higher concentrations of certain target chemicals under high-hydrostatic pressure conditions (e.g., 1,3-diphenylguanidine [DPG]: max. 703 (20 MPa) vs 119 μg/L (0.1 MPa) from cryo-milled tire tread particles under biotic conditions). Under abiotic conditions leaching was weaker for DPG and other chemicals, with contrasting trends for chemicals prone to biotransformation. In crumb rubber leachates chemical concentrations increased with time, but showed no significant differences between biotic/abiotic or high-hydrostatic/atmospheric pressure conditions. Prokaryotic abundance increased in all samples containing tire particles compared to seawater controls, indicating the use of the rubber and/or leached chemicals as an energy source.

## Graphical abstract

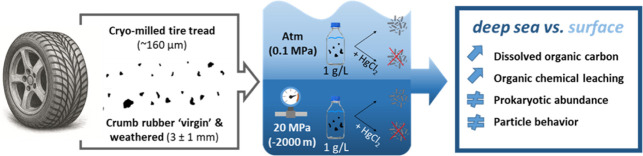

## Introduction

The deep sea is considered a final sink for marine litter, including microplastic, as it will—if not subjected to beaching—eventually sink down in the water column after colonization, biofilm formation or aggregation in marine snow and fecal pellets.^1–4^ Furthermore, it has been suggested that microplastic can be transported from the surface to deeper water layers through ingestion and egestion by migratory species.^5,6^ Once at the seafloor, anthropogenic particles are unlikely to be removed, since natural transport and physical weathering mechanisms (e.g., currents, wind and waves) and chemically degrading mechanisms (e.g., sunlight) are reduced or nonexistent in the deep sea. Indeed, plastic items lost at sea 20 years earlier were reported to be still present in the same area, evidencing that no significant horizontal drifting had occurred.^7^ Plastics can contain high amounts and a large variety of organic and inorganic chemicals,^8,9^ which can be released into the surrounding medium. Little is known on how increased pressure conditions influence the leaching of additives from synthetic materials, which has been shown to be modulated by different environmental factors, such as salinity, temperature, UV exposure or pH.^10–14^ A recent experimental study showed that organic additive release from polyethylene (PE) and soft polyvinyl chloride (PVC) pellets was lower under deep-sea, i.e., high-hydrostatic pressure conditions compared to atmospheric pressure conditions.^15^ For other polymer materials, including elastomers, this information is still lacking.

Tire wear particles (TWP) constitute one of the main sources of anthropogenic particles to the environment, with an estimated emission of 6 million tons per year worldwide^16^ and a global emission per capita of 0.23 to 1.9 kg/year.^17^ Like other microplastics, TWPs contain a variety of organic and inorganic additive compounds, which can leach out of the particles.^18–20^ For example, 1,3-diphenylguanidine (DPG) is used as a vulcanization accelerator in the production of tires and has been identified in tire leachates.^21^ In water samples from an urban watershed receiving road runoff, it reached highest concentrations during rain events (max. 0.52 μg/L)^22^ and in TWP leachate toxicity studies DPG was correlated with deformities in fathead minnow embryos (*Pimephales promelas*).^23^ Furthermore, 6-PPD-quinone (6-PPDQ), a transformation product of the antioxidant *N*-(1,3-dimethylbutyl)-*N′*-phenyl-*p*-phenylenediamine (6-PPD) frequently used in tires, has triggered important research interest after it was discovered in 2021 to induce acute mortality in coho salmon in an urban watershed.^24^ A recent study reported 6PPD-Q concentrations from 0.002 to 0.29 μg/L in stormwaters from the United States.^25^ A study analyzing urban snow from roads and background sites detected 6-PPD at concentrations from 65 to 783 ng/L, 6-PPDQ at 110 to 428 ng/L and DPG at 3 to 14 μg/L.^26^

While some data are available on the occurrence of TWP in surface waters, sediments, road dust and airborne dust,^27–30^ the occurrence of TWP in the deep sea has not been investigated so far. However, a recent study reported the detection of 6-PPD and 6-PPDQ in deep-sea sediments (water depth 1000–2000 m) of the South China Sea, suggesting that emissions of tire-related chemicals have reached deep-sea environments.^31^

An increasing amount of literature on TWP leachates has become available^32–37^ as well as on the influence of environmental factors on the leaching behavior,^18,20,38,39^ but no data is available concerning the potential effects of increased pressure conditions as found in the deep sea. We hypothesized that the specific conditions encountered in deep-sea environments, in particular the high-hydrostatic pressure conditions and the natural prokaryotic assemblages, can have an impact on the leaching of organic chemicals from tire particles, resulting in either increased or decreased amounts of leached compounds. Therefore, our objective was to study the leaching behavior of tire particles (TP) when submerged in (a) seawater collected at the sea surface (1 m depth) and kept under atmospheric pressure conditions (0.1 MPa) and in (b) deep-seawater collected at 2000 m depth and kept under high hydrostatic pressure conditions (20 MPa). Furthermore, biotic and abiotic conditions were applied to study the influence of natural prokaryotic assemblages on the leaching and vice versa. Concentrations of inorganic nutrients and dissolved organic carbon (DOC) were additionally monitored during the experiment.

## Materials and Methods

### Chemicals

All chemicals were of analytical grade and used as received without further purification. Methanol, acetonitrile, and formic acid for UPLC-MS analysis were provided by Biosolve BV (Valkenswaard, Netherlands) whereas ultrapure water was obtained from a Merck Milli-Q Integral 5 system (Merck, Darmstadt, Germany). All standards used are listed in Supporting Information Table S2, with purity grade and supplier information.

### Test Material

Three types of test material were used for this study and have previously been described in a study on chemical leaching from TP under artificial sunlight exposure.^20^ The “cryo-milled tire tread”, CMTT, comprises a mix of tire tread from 20 different tire types (see Table S1 in the Supporting Information for details), which were homogenized through ball milling in cryogenic conditions (Mixer Mill MM 400, RETSCH, Germany). CMTT has been demonstrated to serve as a proxy for tire and road wear particles (TRWP) and allows to study the leaching and transformation of relevant additive chemicals.^39^ However, the organic content in CMTT is approximately 1.8 times higher than in TRWP, resulting in a reduced density (1.2 g/cm^3^ for CMTT versus 1.8 g/cm^3^ for TRWP).^18^ This difference can be attributed to the fact that TRWP are formed in real-world conditions where tire rubber is mixed with road minerals, asphalt, and brake dust, and undergoes thermal and oxidative degradation, whereas CMTT is lab-generated from pure tire tread and contains mostly undamaged organic rubber. Further, the CMTT median particle diameter is expected to be bigger (∼160 μm) than in real TRWP (∼70 μm).^18^ The size distribution and particle shape images of our CMTT are provided in Figure S1.

The two other test materials were made of crumb rubber granulate as used in artificial sport fields. The original material (hereafter called “virgin crumb rubber”, VCR) made from end-of-life tires was sourced from a sports field in Tromsø (Norway) prior to application on the field and has previously been used for leaching and toxicity testing.^19,33^ Part of the crumb rubber was exposed in situ to natural seawater in Tromsø (hereafter called “weathered crumb rubber”, WCR) to create a test material of high environmental relevance. Five grams of VCR particles were placed in cylindric meshed stainless-steel containers (Inoxia, UK) and submerged in marine surface waters (3–4 m depth). The particles were placed in the container with sufficient headspace, allowing them to move freely when immersed in seawater. During exposure, seawater temperature ranged from −2.15 to 13.9 °C (average 6.44 °C). After recovery, the WCR was not modified (i.e., no removal of the biofilm or other cleaning) prior to use but stored in dry and dark conditions for approximately 1 year. Due to the limited amount of available test material different batches of WCR exposed in situ for 12–18 months were homogenized for this study.

### Sea Water Collection

Deep-seawater (DW) was collected during the EMSO-LO cruise^40^ on the French R/V “*Pourquoi Pas?”* on February 10, 2022, at 2000 m depth, 30 km off the coast of the French mainland in the western Mediterranean Sea (42.80242 N, 5.98674 E). Typical for the Mediterranean basin, the sampling station, which has a total depth of about 2400 m, was characterized by for winter conditions relatively high temperatures (around 13 °C) throughout the water column (Figure 1). In addition, surface water (SW) was collected at a depth of 1 m. DW and SW seawater was collected using polytetrafluoroethylene (PTFE)-coated GO-FLO sampling bottles (General Oceanics, USA) mounted on a rosette sampler and remotely closed by messenger. A CTD-logger (conductivity–temperature-depth) was used to collect relevant metadata throughout the water column (Figure 1). Once retrieved, the outlet of the GO-FLO bottles was opened to evacuate the first milliliters of seawater, before pouring the remaining water into a glass bottle. As water from several GO-FLO bottles was needed, a 10 L glass bottle was used for homogenization.

**Figure 1:**
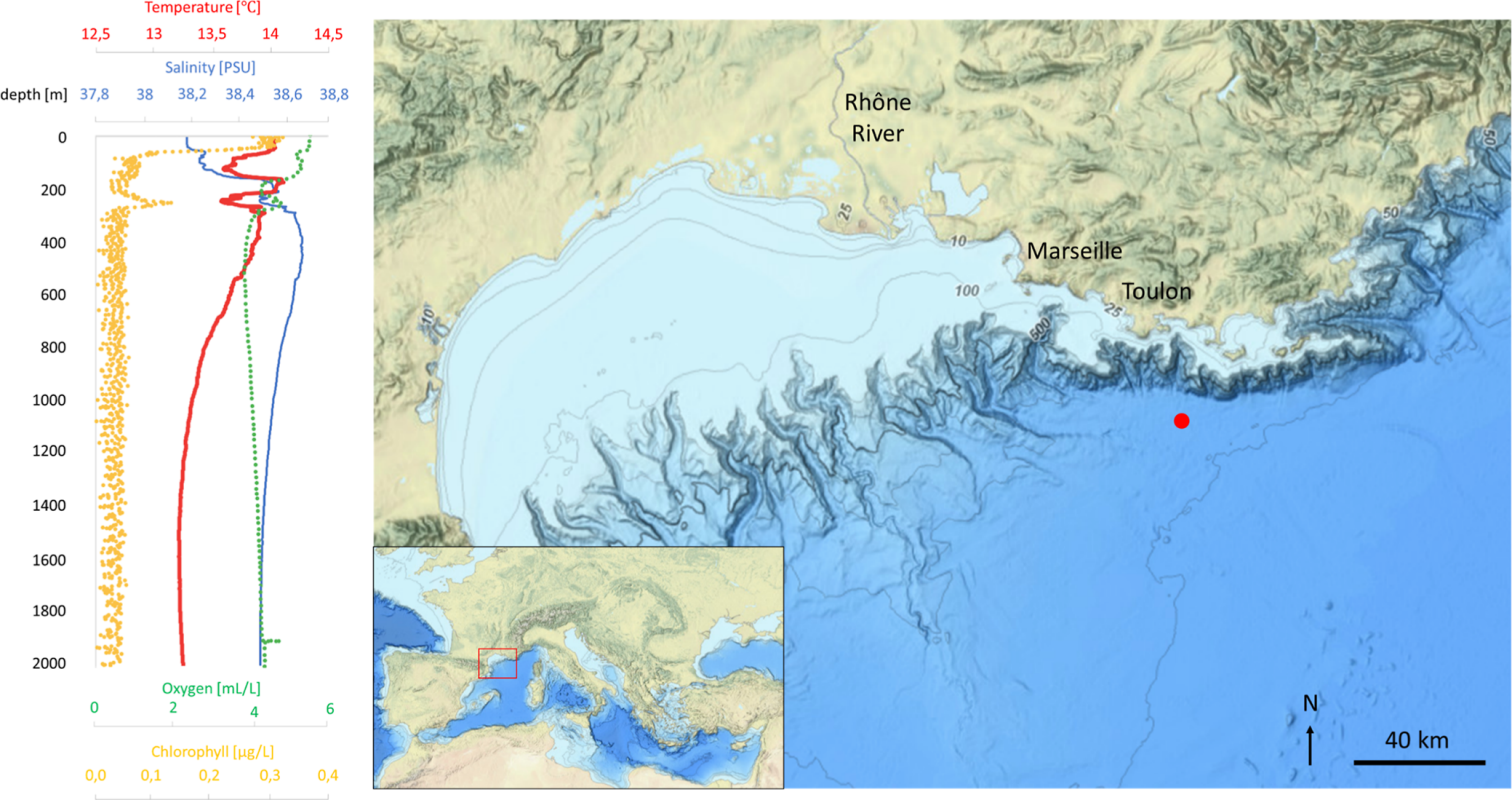
: Temperature, salinity, oxygen and chlorophyll a profile of the sampling station (left). Map indicating the position of the sampling station (red dot) in the Northwestern Mediterranean Sea (right). Map source: National Oceanic and Atmospheric Administration (public domain).

### Hydrostatic Pressure Experiment

The seawater samples were then filtered onboard through precombusted 0.7 μm, GF/F filters (WHATMAN) using a glass filtration unit and filled into precombusted 100 mL Schott bottles (Duran, DWK Life Sciences GmbH, Germany) containing the test material at a concentration of 1 g/L. This dispersion concentration was applied to all three test materials. The Schott bottles were filled up completely to avoid the presence of air bubbles, leading to a final volume of approximately 130 mL. To create abiotic conditions, a subset of samples was poisoned using mercuric chloride (HgCl_2_, final concentration 10 mg/L). This approach has previously been used to create abiotic conditions in leaching experiments as well as chemical degradation studies^11,15,41^ and is not expected to have a major impact on the leaching itself. However, minor impacts on individual target compounds cannot be excluded at this stage and would require further testing. The experimental design is depicted in Figure 2.

**Figure 2:**
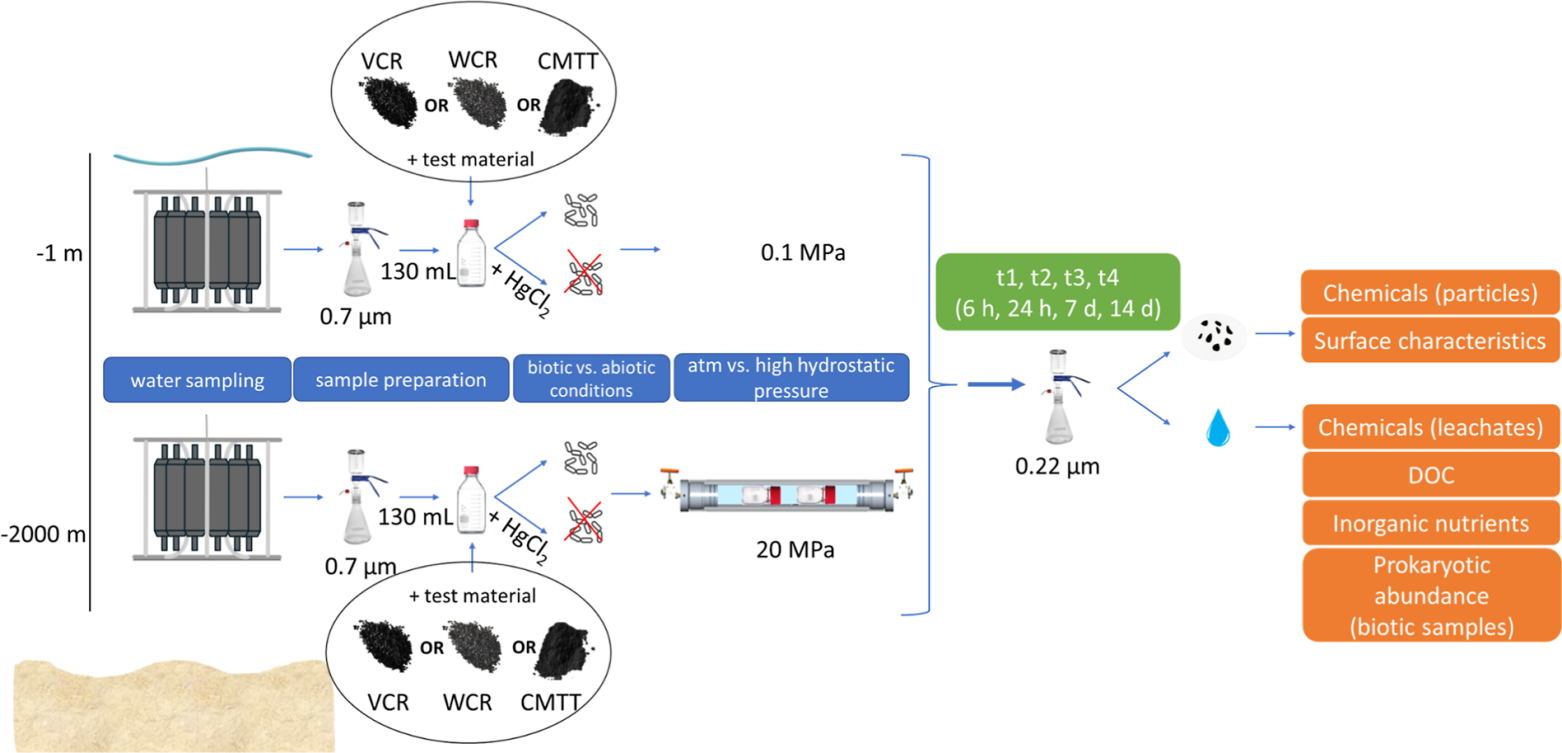
: Experimental design indicating the strategy for water sampling preparation, experimental conditions, sampling time points and analysis performed on test material (particles) and leachates.

The DW samples were subjected to a pressure of 20 MPa using 500 mL high-pressure bottles (HPB-500, Top Industrie, Vaux-le-Pénil, France) and a piloted pressure generator.^42,43^ For such an aim, two 100 mL-Schott bottles closed with a modified plastic screw cap including a rubber septum coated with PTFE were inserted into an HPB-500 (Figure 2). The hydrostatic pressure applied to the HPBs was transmitted to the samples via the septum of the Schott bottles. In contrast, the SW samples were kept in the dark under atmospheric pressure conditions (0.1 MPa). All samples were kept in a temperature-controlled room at 13 ± 1 °C, representing in situ seawater temperature conditions (see Figure 1). Triplicate samples of each treatment (SW biotic, SW abiotic, DW biotic and DW abiotic) and each test material (CMTT, VCR, WCR) were sacrificed for analysis after 6 h, 24 h, 7 and 14 d of exposure. Each sample consisted of one Schott bottle. Once incubation was complete, the hydrostatic pressure in the HPB-500 bottle was slowly reduced using the pilot-operated pressure generator. Then, the HPB-500 bottle was opened and all Schott bottles contained within this HPB-500 bottle were sacrificed. Due to a limited amount of material available for the experiment, abiotic time points *t*_1_–*t*_3_ (6 h, 24 h, 7 d) are lacking for WCR.

### Sample Treatment and Analysis

Each sample was filtered through a 0.22 μm poly­(ether sulfone) (PES) filter (Millipore Express PLUS, Merck KGaA, Darmstadt, Germany) to remove the test material and reduce microbial activity during sample storage. The retention of target compounds on the filter was tested and related recoveries are reported in Figure S2. The filters were then wrapped in precombusted aluminum foil and stored in the dark at 4 °C. At the end of the experiment (14 days), particles retained on the filter were extracted with methanol using an ultrasonic bath for 1 h, following a procedure previously described.^44^ The aqueous phase was divided into subsamples as follows: 30 mL were collected for chemical analysis using ultraperformance liquid chromatography time-of-flight mass spectrometry (UPLC-TOF-MS), stored in glass vials in the dark at 4 °C, and subsequently analyzed by direct injection without prior extraction or concentration. Fifteen mL were collected for DOC analysis, acidified (with 85% H_3_PO_4_ acid, final pH ∼ 2) and stored in precombusted glass vials in the dark at 4 °C until analysis using a Shimadzu TOC-5000 (Shimadzu Co., Japan) carbon analyzer (see Supporting Information for more information). For DOC analysis, only one replicate per time point was considered. For the determination of prokaryotic abundance in samples from biotic conditions, 1.98 mL of sample were collected and mixed with glutaraldehyde (0.25% final) and poloxamer (0.01% final), and stored at −80 °C until analysis.^45^ Samples were analyzed using the CytoFLEX (Beckman Coulter, Inc., USA) of the PRECYM flow cytometry platform (https://precym.mio.osupytheas.fr/). For heterotrophic prokaryote enumeration, samples were labeled with SYBR Green II solution (Molecular Probes, USA, commercial solution diluted 10 times), to stain the nucleic acids. Combining SYBR Green II fluorescence and light diffusion parameters allowed to distinguish unambiguously the cells from inorganic particles, detritus and free DNA.^46^ For the analysis of NO_3_^–^ and PO_4_^3–^, 20 mL of the filtered sample were filled into PP (polypropylene) tubes previously rinsed three times with a sample aliquot and kept in the dark at −20 °C until analysis. Nitrate and phosphate concentrations were measured by colorimetric methods using an automated AA3 AutoAnalyser (SEAL Analytical, USA).

### Analysis of Target Chemicals

A total of 17 tire related compounds were quantified in the aqueous leachates using UPLC-TOF-MS: 1,3-diphenylguanidine (DPG), phenylguanidine (PG), triphenylguanidine (TPG), 2-(methylthio)­benzothiazole (2-MTBT), 2-hydroxybenzothiazole (2-OHBT), 2-aminobenzothiazole (NH2-BT), benzothiazole-2-sulfonic acid (BTSA), 2-(4-morpholinyl)­benzothiazole (2-(4-Mo)-BT), *N*-(1,3-dimethylbutyl)-*N*-phenyl-1,4-phenylenediamine (6-PPD), 6-PPD-quinone (6-PPDQ), aniline, 4-hydroxydiphenylamine (4-HDPA), *N*,*N*-diphenyl-*p*-phenylendiamine (DPPD), *N*-nitrosodiphenylamine (NO–DPA), dicyclohexylamine (DCH), *N*-methyldicyclohexylamine (MeDCH) and hexa­(methoxymethyl)­melamine (HMMM). These compounds consist of tire-related additives (such as e.g. vulcanization accelerators and antioxidants) as well as some of their transformation products. These substances were chosen based on their well-established association with tire rubber and their prior identification and quantification in the same test materials used in this study, as previously reported.^20^ These were grouped into the following chemical classes for the discussion of results: *phenylguanidines* (DPG, PG, TPG), *benzothiazoles* (2-MTBT, 2-OHBT, NH2-BT, BTSA, 2-(4-Mo)-BT), *phenylenediamines* (6-PPD, 6-PPDQ, 4-HDPA, DPPD, NO–DPA), and *other amines* (DCH, MeDCH, HMMM, aniline).

In spiked seawater samples, four compounds showed recoveries below 70% (6-PPD, DPPD, HMMM, and aniline). Notably, DPPD recovery was strongly affected by retention on the PES filters, with values of only 10–15% compared to 90–100% in the absence of filters. To maintain methodological consistency across all 17 analytes, results were not recovery-corrected. While this may lead to underestimation for certain compounds, full recovery data are provided to ensure transparency and facilitate interpretation (Figure S2). LOD and LOQ values were defined based on a signal-to-noise ratio greater than 3 for LOD and greater than 10 for LOQ using an external calibration curve (Table S2). Instrumental method details can be found in the Supporting Information (Text S1).

### Scanning Electron Microscope Analysis

For scanning electron microscopy (SEM) dried particles were scattered onto standard SEM stubs with sticky-carbon-pads (Plano GmbH, Germany). The samples were then blown with compressed nitrogen to remove loose particles. To make the surface of the particles electrically conductive in order to avoid image-distortion and oversaturation caused by charging under the electron-beam, the samples were sputter-coated with a thin layer (∼30 nm) of gold–palladium (80%/20%) using a Leica SCD500 sputter-coater (Leica Microsystems, Wetzlar, Germany). Imaging was carried out with a Zeiss Merlin VP Compact field-emission SEM (Carl Zeiss Microscopy, Oberkochen, Germany). The electron acceleration energy amounted to 5 kV, the beam current was approximately 250 pA. To obtain strong topographic contrast, secondary electrons were detected using an Everhard-Thornley-type detector.

### QA/QC

All glassware was combusted (450 °C, 6 h) prior to use and covered with combusted aluminum foil. Plastic and rubber materials were avoided whenever possible. Apart from the water filtration onboard the research vessel, all sample processing steps were conducted in an ISO class 6 clean room equipped for particle removal for organic trace analysis. Cotton lab coats and nitrile gloves were worn during sample handling. Control samples (Table S6) containing only filtered seawater were made for all experimental conditions and time points (with triplicate control samples for starting and final time points t0 and t4) and kept under the same temperature and pressure conditions as samples containing test materials.

### Statistical Analysis

To evaluate the significance of differences between different experimental conditions, a *t*-Test: Paired Two Sample for Means was performed using Microsoft Excel (Microsoft Corporation, Redmond, WA, USA). Data from the two related conditions were organized in adjacent columns, with each row representing a matched pair of observations. The analysis was conducted using the built-in *Data Analysis ToolPak*, which provided the mean difference, t statistic, degrees of freedom, and associated two-tailed *p*-value. Differences were considered statistically significant at *p* < 0.05.

## Results and Discussion

### Inorganic nutrients

The Mediterranean is an oligotrophic sea characterized by low nutrient concentrations and high solar radiation.^47^ Generally, deep-sea waters here exhibit overall higher concentrations of oxidized forms of inorganic nutrients, such as NO_3_^–^ and PO_4_^3–^, than surface waters.^48^ In our study, we confirmed differing characteristics of SW and DW samples: NO_3_^–^ concentrations were higher in DW samples (mean 8.13 ± 1.05 μM, all DW samples combined) compared to SW samples (mean 0.52 ± 0.06 μM).

For PO_4_^3–^, we could furthermore distinguish differences depending on the test material present in the samples. The DW samples exhibited a mean PO_4_^3–^ value of 0.39 ± 0.07 μM (excluding WCR) and the SW samples a concentration of 0.03 ± 0.01 μM (excluding WCR). In samples containing WCR this mean concentration was notably higher: 1.20 ± 0.22 μM in DW and 0.87 ± 0.12 μM in SW samples, representing an increase of approximately 0.8 μM in both types of water samples (Figure S3). The PO_4_^3–^ was likely released from a biofilm, formed during the previous stay in seawater, which was intentionally not removed prior to the experiment. Residues of a former biofilm were observed on WCR particles using SEM imaging, likely formed during the natural weathering of the material, as similar residues were not detected on the corresponding virgin material (VCR) (Figure S4).

### Prokaryotic Abundance

Generally, an increase in the abundance of heterotrophic prokaryotes was observed for t_3_ and t_4_ samples, i.e. after 7 and 14 days of exposure, respectively (Figure 3). This is also true for seawater control samples, confirming that our experimental setup allowed microbial growth in biotic samples. When comparing the test materials, highest abundances were observed in WCR samples, followed by VCR when considering SW conditions, but by CMTT when considering DW conditions. This suggests that the remains of a previous biofilm on WCR particles provided an additional source of nutrients for the prokaryotes to promote growth, compared to the nonweathered VCR. This is in accordance with the PO_4_^3–^ data (see section “*Inorganic nutrients*”), suggesting a release of PO_4_^3–^ from the biofilm into the water phase, followed by a consumption of PO_4_^3–^ through prokaryotes, translating into an increase in the prokaryotic abundance. The biofilm was furthermore a source of organic carbon, as reflected in the DOC measurements (see section on DOC data), which likely also promoted prokaryotic growth.

**Figure 3:**
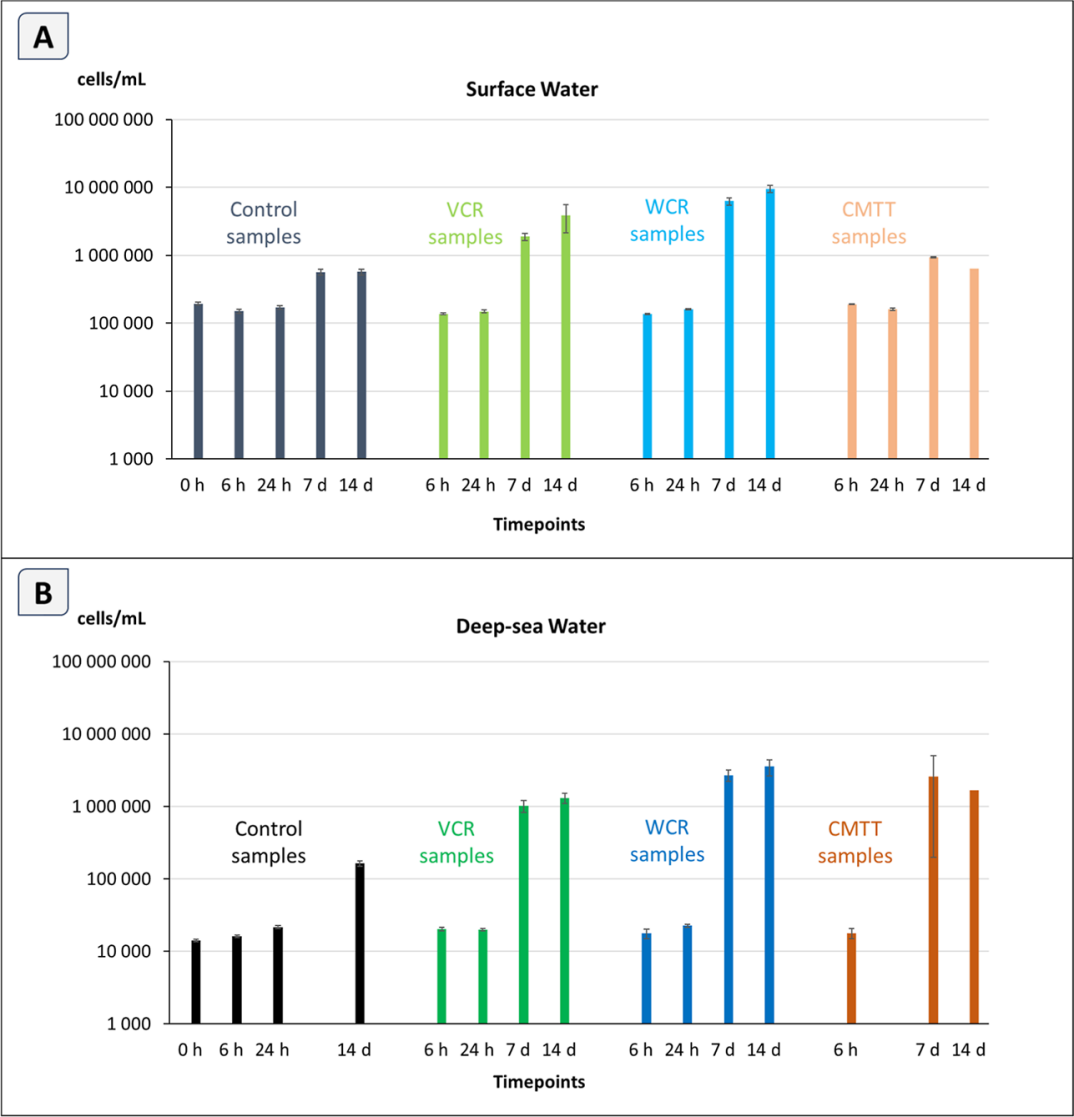
: Mean prokaryotic abundance in cells per milliliter (cells/mL) in surface seawater (A) and deep-seawater (B) samples. Note that DW t_3_ (7 days) control and t_2_ (24 h) CMTT samples were not available and CMTT t_4_ (14 days) samples were not analyzed as replicates. Error bars represent standard deviations calculated from true triplicates. Note the logarithmic *y*-axis. Control samples represent seawater collected, handled and stored under the same experimental conditions as real samples, but without the addition of test material.

DW samples exhibited lower prokaryotic abundances at the beginning of the experiment (*t*_0_) as expected,^48^ but the prokaryotic abundance increased for all test materials (Figure 3).

In CMTT samples a higher prokaryotic abundance was observed in DW samples compared to SW samples. This might be due to a change in the state of aggregation of the CMTT particles when submitted to increased pressure: in SW samples the particles exhibited a hydrophobic behavior and thus stayed at the very surface of the water layer (contact area between the water and the bottle cap); this may have limited the contact between the water and the particles and, thus, the leaching of tire constituents. This is supported by the DOC data, which shows lower concentrations in SW samples for CMTT compared to DW samples (Table S3). Under high-hydrostatic pressure conditions, however, the CMTT particles aggregated and sank to the bottom of the sample bottles (Figure S5). This difference in the spatial distribution and state of agglomeration of CMTT probably impacted the leaching of chemicals as well as the availability of the particles as a substrate and energy source for the growth of prokaryotes. However, it should be noted that such behavior may not fully reflect the characteristics of real TRWP, which are typically embedded with mineral incrustations and other road-derived particles. These features contribute to a higher overall density of TRWP (approximately 1.80 g/cm^3^) compared to CMTT (1.20 ± 0.02 g/cm^3^), potentially resulting in more rapid sedimentation even under atmospheric pressure conditions.^18,49^

For VCR and WCR particles no changes in aggregation were observed and the particles settled at the bottom of the glass bottles under both pressure conditions. Consequently, each of the three test materials had a different impact on the abundance of heterotrophic prokaryotes, highlighting the complexity of these interactions and the need to include different test materials (shapes, sizes, degree of weathering) in laboratory studies examining e.g. biodegradation or microplastics as vectors for microorganisms.

### Particle Characterization

In order to investigate the presence of remaining biofilm or microbes on the particle surface after the experiment, scanning electron microscope (SEM) was used on the dry particles. As a result, residuals of a biofilm were found on the WCR surface on particles from both abiotic and biotic experimental samples (Figure S4), confirming the hypothesis that organic material was deposited on the surface of the particles, and microorganisms settled on the particles during the natural weathering prior to the experiment. This likely influenced both the inorganic nutrients and prokaryotic abundance values discussed in the dedicated sections above. Prokaryotic colonies were clearly visible on the surface of VCR particles from biotic condition samples (Figure S4), suggesting that the nonweathered material was acting as a fresh carbon source which had been used as a substrate for prokaryotic growth. On the contrary, no visible prokaryotes were identified on the surface of CMTT particles. This might be attributed to the irregularity of the surface of these particles (Figure S4), whose presence of lumps and agglomerates might mask remaining prokaryotic cells or to a limited amount of test material observed using SEM.

### Inventory of Dissolved Organic Carbon

The DOC concentrations measured in the surface (51.5 ± 3.9 μM or 0.62 ± 0.05 mg/L) and deep-seawater (41.1 ± 4.9 μM or 0.49 ± 0.06 mg/L) control samples are consistent with values previously reported in literature, where the deep sea is generally characterized with a lower DOC content.^50^

The presence of test material in the samples led to an increase in DOC concentrations in all cases, indicating a leaching of organic chemicals and/or black carbon from the particles into the seawater (Figure 4a and Table S3). As in a previous leaching study using the same three test materials,^20^ CMTT released the highest amounts of DOC (max. 175 μM or 1.7 mg/L or 1.7 mg/g CMTT) compared to WCR (max. 105 μM or 0.8 mg/L) and VCR (max. 95 μM or 0.6 mg/L). DOC concentrations could potentially be influenced through nanoparticles <0.22 μm present in the CMTT and/or generated during the exposure experiment, though it is unknown if these particles could pass through the micropores of the filters or would rather agglomerate and be retained.

**Figure 4:**
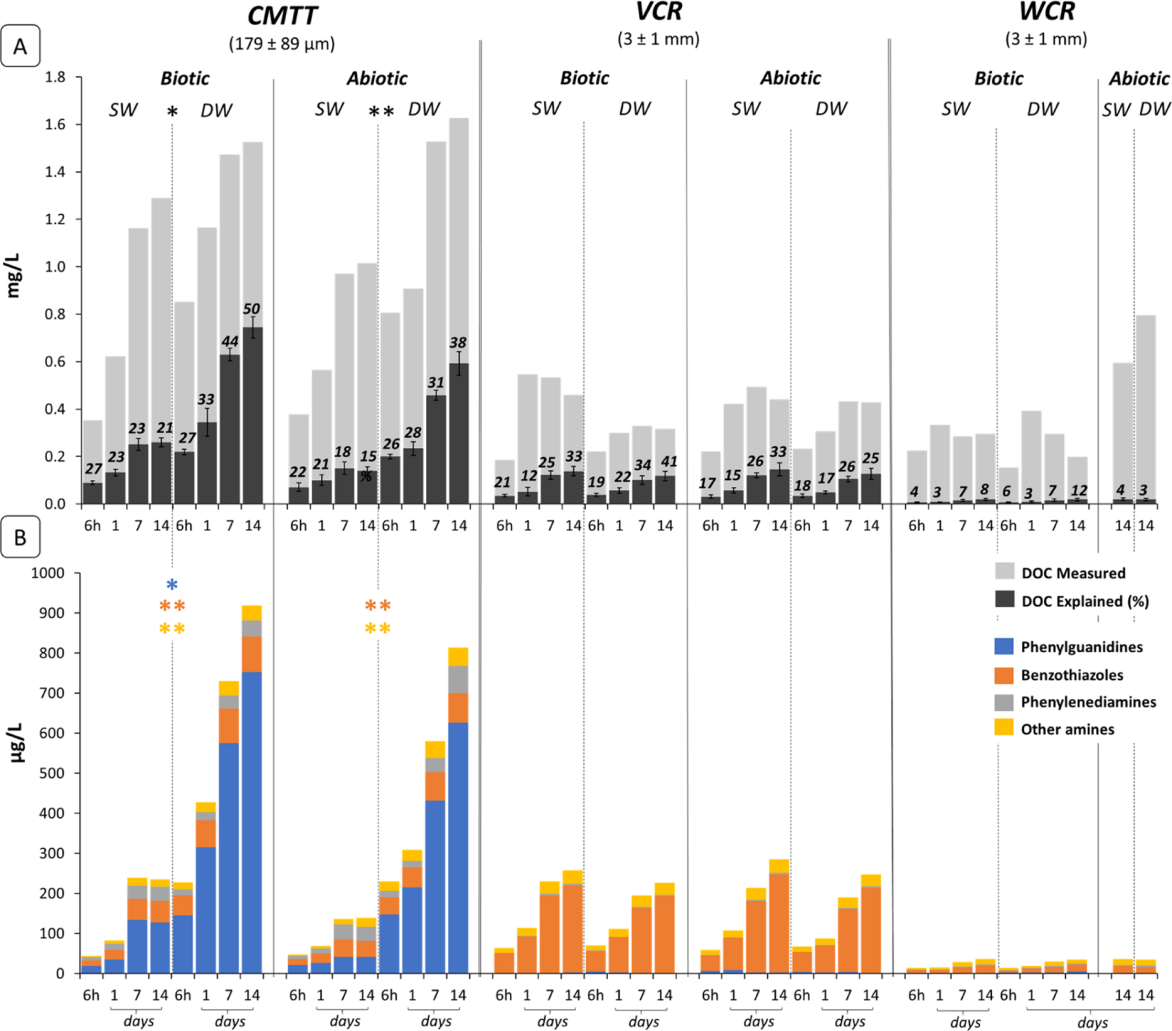
: Measured and explained dissolved organic carbon (DOC) (A) and the measured concentrations of targeted analyte groups used to calculate explained DOC (B) for cryo-milled tire tread mixture (CMTT), virgin and weathered crumb rubber (VCR & WCR, respectively), of both biotic and abiotic conditions at atmospheric (surface water “SW”) and high hydrostatic (deep seawater “DW”) pressure. Asterisks indicate significance of differences between SW and DW (*p < 0.05, **p < 0.005); absence of an asterisk denotes a nonsignificant difference. Error bars indicate the standard deviation from 3 independent analyses. Note: In panel (a), explained DOC is calculated based on the carbon content (mol C) of the quantified compounds, not their full molecular mass. Therefore, the total compound classes concentrations shown in (B) are not expected to match the total explained DOC values reported in (A).

In the case of CMTT samples, the DOC measurements demonstrated an influence of high hydrostatic pressure, with higher DOC concentrations compared to atmospheric pressure samples. In contrast, the DOC values for the CR samples (WCR and VCR) did not reflect a similar impact of the hydrostatic pressure. This discrepancy may be attributed to the smaller mean particle size of CMTT (∼160 μm) compared to the larger particle sizes of VCR and WCR (2–4 mm), resulting in a much higher surface-to-volume ratio for CMTT, subsequently favoring the release of dissolved organic compounds. Concentrations in VCR and WCR samples seemed generally similar, though for WCR only a restricted number of abiotic time points was obtainable due to a limited amount of available test material (Figure 4a).

### Contribution of Leached Compounds to DOC

To assess the extent to which measured DOC is explainable by the quantified target compounds, their relative contributions to total DOC was calculated (Figure 4a). In CMTT leachates, samples exposed to high-hydrostatic pressure exhibited an increasing proportion of DOC contribution accounted for by target compounds over time, reaching up to 50% under biotic and 38% under abiotic conditions. In contrast, samples maintained at atmospheric pressure showed markedly lower contributions of target compounds (approximately 23% and 20%, respectively). This suggests that high-hydrostatic pressure primarily enhances the physical release of measurable compounds, making their contribution to DOC nearly as significant as that of unidentified substances. In the case of CR samples, increasing trends in DOC contributions over time were observed in both SW and DW samples. Differences in DOC contribution between biotic and abiotic conditions were also observed, though less pronounced than in CMTT leachates. Under biotic conditions, the DOC contributions were slightly higher in DW than in SW, reaching 41% vs 33% and 12% vs 8% for VCR and WCR, respectively, while in abiotic conditions the DOC contribution was lower in DW compared to SW samples (reaching 23% vs 33% for VCR and WCR, respectively). Interestingly, under all test conditions, a higher contribution of target compounds to DOC was observed under biotic compared to abiotic conditions. This can be attributed to the fact that biotic conditions can enhance the formation of derivatives compounds (such as 2-MTBT, a biotransformation product of 2-MBT), as well as to a likely higher degradability of unidentified precursor compounds which can lead to the formation of target compounds.

### Target Analytes in Cryo-Milled Tire Tread Leachates—Atmospheric vs High-Hydrostatic Pressure Conditions

In CMTT leachates, 16 of the 17 target compounds were detected at concentrations above LOQs under all experimental conditions. In contrast, the concentrations of these compounds in the corresponding control samples remained below the LOQs (Table S6). This suggests that CMTT is a source of a high diversity of organic compounds, under atmospheric pressure as well as under high-hydrostatic pressure conditions. DPG was the most prominent compound found in the CMTT leachate samples, clearly showing the influence of the high-hydrostatic pressure by reaching considerably higher values at 20 MPa in both biotic and abiotic conditions (703 and 584 μg/L, respectively) (Figure 5a and Table 1). Other abundant compounds were benzothiazoles (Figure 5), in particular 2-OHBT and BTSA, reaching highest concentrations under high-hydrostatic pressure (around 40 and 20 μg/L, respectively) compared to atmospheric pressure (around 30 and 4 μg/L, respectively) (Table 1 and Figure 5c).

**Figure 5:**
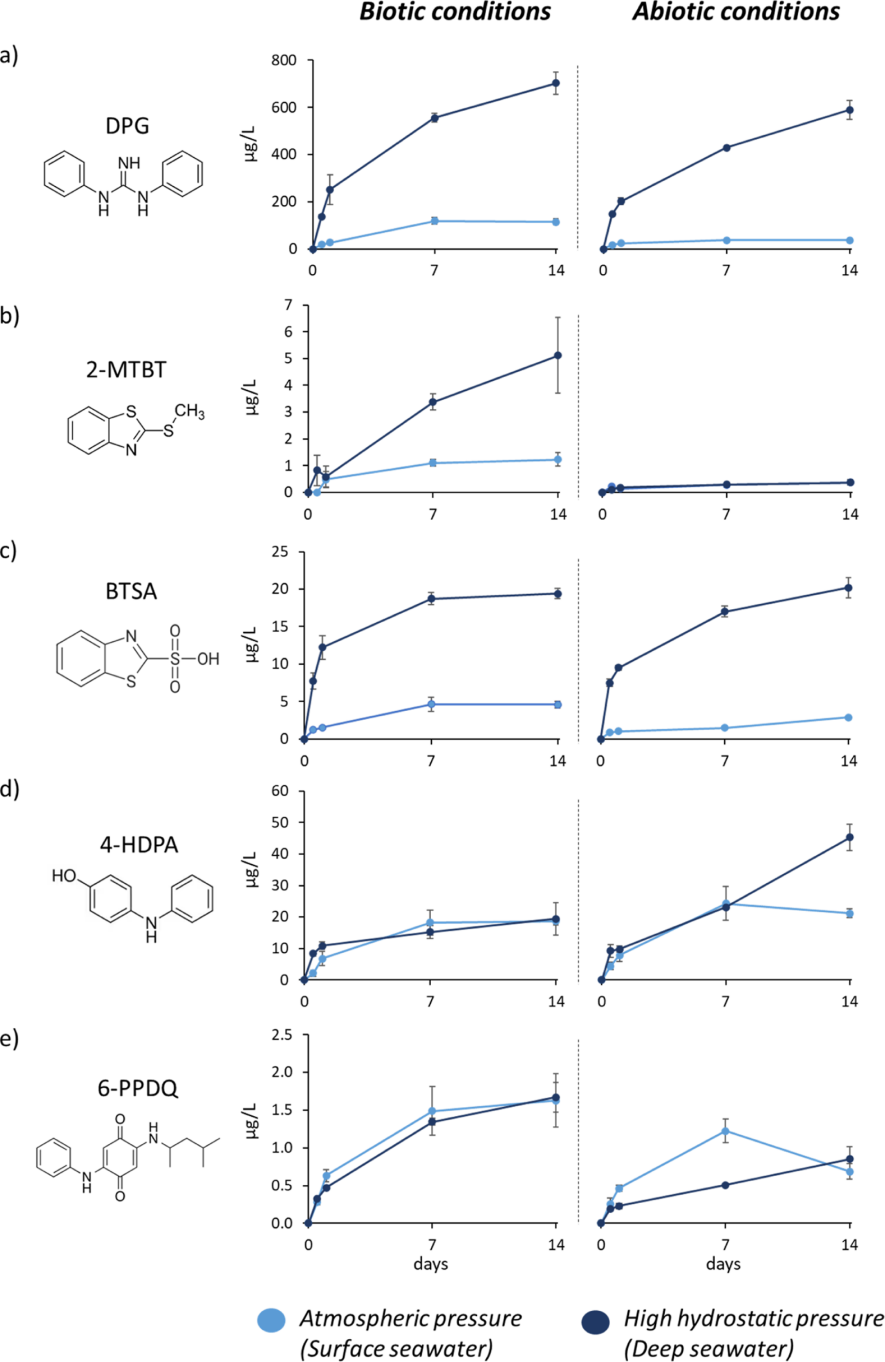
Concentrations of 1,3-diphenylguanidine (DPG) (a), 2-methylthiobenzothiazole (2-MTBT) (b), benzothiazole-2-sulfonic acid (BTSA) (c), 4-hydroxydiphenylamine (4-HDPA) (d), and 6-PPD-quinone (6-PPDQ) (e) in both biotic and abiotic conditions at atmospheric (0.1 MPa, light blue lines) and high-hydrostatic (20 MPa, dark blue lines) pressure for CMTT samples. Error bars indicate the standard deviation from 3 independent replicates.

**Table 1:** : Concentrations (±SD, *n* = 3) of TWP Chemicals in the Seawater Leachates at the End of the Aging Experiment (day 14) of the Cryo-Milled Tire Tread Mixture (CMTT) Samples

compound	abbreviation	biotic conditions		abiotic conditions	
		atmospheric pressure (0.1 MPa)	high-hydrostatic pressure (20 MPa)	atmospheric pressure (0.1 MPa)	high-hydrostatic pressure (20 MPa)
		μg/*L* (=μg/*g*_CMTT_)			
*phenylguanidines*					
1,3-diphenylguanidine	DPG	114 (±13)	703 (±47)	37 (±4)	584 (±40)
phenylguanidine	PG	0.16 (±0.01)	0.97 (±0.06)	0.13 (±0.05)	0.94 (±0.18)
triphenylguanidine	TPG	0.37 (±0.03)	0.65 (±0.05)	0.38 (±0.05)	0.54 (±0.07)
*benzothiazoles*					
2-methylthiobenzothiazole	2-MTBT	1.2 (±0.2)	5.1 (±1.4)	0.4 (±0.1)	0.4 (±0.1)
2-hydroxybenzothiazole	2-OHBT	33 (±2)	45 (±2)	23 (±5)	36 (±4)
2-aminobenzothiazole	NH2-BT	0.18 (±0.02)	0.37 (±0.02)	0.14 (±0.03)	0.28 (±0.05)
benzothiazole-2-sulfonic acid	BTSA	4.6 (±0.5)	19 (±0.7)	2.9 (±0.2)	20 (±1.4)
2-(4-morpholinyl)-benzothiazole	2-(4-Mo)-BT	10 (±1)	14 (±1)	8 (±1.5)	12 (±0.4)
*phenylenediamines*					
*N*-(1,3-dimethylbutyl)-*N*-phenyl-1,4-phenylenediamine	6-PPD				
4-hydroxydiphenylamine	4-HDPA	19 (±1)	19 (±5)	21 (±1)	45 (±4)
6-PPD-quinone	6-PPDQ	1.6 (±0.4)	1.7 (±0.2)	0.7 (±0.1)	0.9 (±0.2)
*N*,*N*-diphenyl-*p*-phenylendiamine	DPPD	0.18 (±0.03)	0.15 (±0.03)	0.42 (±0.03)	0.57 (±0.05)
*N*-nitrosodiphenylamine	NO–DPA	0.35 (±0.02)	0.35 (±0.05)	0.26 (±0.02)	0.66 (±0.13)
*other amines*					
aniline	aniline	11 (±2)	12 (±1)	9 (±2)	13 (±3)
dicyclohexylamine	DCH	15 (±1)	30 (±2)	15 (±3)	34 (±3)
*N*-methyldicyclohexylamine	MeDCH	0.8 (±0.07)	2.0 (±0.2)	0.8 (±0.2)	2.0 (±0.3)
hexa(methoxymethyl)melamine	HMMM	1.3 (±0.5)	2.5 (±0.7)	3.4 (±0.6)	7.1 (±0.1)

Generally, the concentration of target *phenylguanidines*, *benzothiazoles* and *other amines* was notably higher (from 50% to over 100%) in DW compared to SW samples in both biotic and abiotic conditions (Figure 4b). This could be attributed to the fact that the high pressure allowed the water to penetrate deeply into the particle’s interstices, as the particles subjected to high-hydrostatic pressure sank to the bottom of the vessel (Figure S5). Furthermore, by performing a solvent extraction of the CMTT particles after the aging, a lower amount of extractable compounds was obtained in the DW compared to the SW particles, indicating that the increased chemical leaching which occurred under high-hydrostatic pressure decreased the concentration remaining in the CMTT particles (Figure S6). Indeed, it is likely that under high-hydrostatic pressure, the polymer matrix might compress, reducing its volume. This compression could squeeze the physically entrapped additives out of the polymer matrix, especially from polymers with high elasticity, such as elastomers used in tire particles. Further research on the mechanisms behind pressure-induced chemical leaching would, however, be needed to confirm this hypothesis.

In contrast, most of the *phenylenediamines* exhibited a similar behavior in both surface and deep seawater condition leachates, which can be attributed to the fact that the water solubility of these compounds is much lower and their reactivity higher than of the other compound classes considered.^18^ Additionally, these compounds are known to migrate within the rubber matrix to enhance durability and degradation resistance.^51^ This inherent mobility suggests they are less tightly bound to the polymer structure, potentially making them more accessible at or near the surface. As a result, elevated pressure may have a limited effect on their leaching, since they are not deeply embedded within the matrix.

### Target Analytes in Cryo-Milled Tire Tread – Biotic vs Abiotic Conditions

For *phenylguanidines* and *benzothiazoles*, slightly higher concentrations were observed under biotic than abiotic conditions, which might be attributed to the prokaryotic activity, which has been shown to promote the release of plastic additives.^11,15^ Additionally, in the case of *benzothiazoles*, the increased concentrations under biotic conditions may also result from the formation of biotransformation products, such as 2-MTBT], a bacterial methylation product of the vulcanization accelerator 2-MBT.^52^ 2-MTBT reached concentrations of 5.1 μg/L in biotic DW samples compared to 0.4 μg/L in abiotic DW samples (Figure 5b), underlining the impact of biological activity.

For *phenylenediamines* and *other amines* no major differences between biotic and abiotic conditions were observed. Only 4-HDPA, a hydrolysis product of 6-PPD commonly found as a TWP related contaminant,^37^ showed a >100% increase in abiotic DW samples compared to the biotic ones (Figure 5d). This may suggest that 4-HDPA was degraded by prokaryotes.^53^ A contrasting behavior was observed for 6-PPDQ, one of the oxidation products of 6-PPD,^24,37^ for which a 100% increase was observed in biotic samples compare to the abiotic ones at day 14 (Table 1 and Figure 5e), indicating that oxidation processes were stronger due to the presence of biological activity. While we are not aware of a study investigating this phenomenon in marine waters, it has been shown that microbial activity in soils lead to an increased oxidation of 6-PPD, suggesting a microbial contribution to the formation of 6-PPDQ.^54^ 6-PPD itself was only detected at levels <LOQ in our study (Table S4), suggesting rapid transformation.

Our results indicate that prokaryotic activity strongly modulates the fate of organic chemicals from tire material in marine waters through transformation and degradation processes. All data on leached chemicals in CMTT samples can be found in Table S4 of the Supporting Information.

### Crumb Rubber Leachates

Regarding both virgin and weathered crumb rubber (VCR & WCR) samples, an increasing trend in chemical concentrations was observed in the aqueous leachates over time (Table S5, Supporting Information). Final concentrations were more than 100% higher than the initial values. However, no clear differences were observed between SW and DW conditions, probably due to the larger size of the VCR and WCR particles (between 2 and 4 mm) compared to CMTT (∼160 μm), which led to a reduced impact of the hydrostatic pressure in the leaching of chemicals. Indeed, as small particles such as CMTT present a higher surface area/volume ratio, a greater proportion of the particles’ material (including the additives) is exposed to the surrounding environment. This facilitates very likely more extensive interaction between the water and the additives, enhancing the dissolution or leaching process.

Of the compounds analyzed, benzothiazoles were predominant in VCR samples and occurred in higher concentration than in the CMTT samples (Figure 4b). Lower concentrations of *phenylguanidines*, *benzothiazoles* and *other amines* were found in WCR compared to VCR leachates, as expected due to the long environmental aging which the WCR particles underwent before this experiment, which agrees with previous works.^20^ However, the summed concentrations of *phenylenediamines* were comparable between VCR and WCR samples, suggesting a slow release of this chemical reservoir from the tire particles regardless of long-term weathering. As the generally low concentrations of leached target compounds in WCR samples are unlikely to explain the high prokaryotic abundance observed in these samples, we assume that the organic residuals present on the particles after the natural weathering might have promoted the prokaryotic growth.

While WCR released substantially fewer chemical amounts into seawater than VCR (∑_17_ max. 29.8 vs 240 μg/L, respectively), consistent with weathering-induced surface depletion, solvent extraction of both materials yielded similar total chemical loads (Figure S6). This suggests that environmental weathering primarily reduced the aqueous accessibility of surface chemicals—for example via surface oxidation (e.g., formation of an oxidized surface layer that impedes diffusion) or biofilm-mediated retention—rather than depleting the bulk reservoir of extractable compounds.

All data on leached chemicals in VCR and WCR samples can be found in Table S5 of the Supporting Information.

### Comparison with Previous Works

A previous study using a similar experimental setup found the presence of PVC particles to decrease the number of prokaryotes over time in SW and DW samples compared to controls, indicating a potential toxic effect of the PVC leachate.^15^ This is in contrast to our observations, highlighting that different polymers and their leachates can have highly differing impacts on marine prokaryotes. In both studies (conducted by the same laboratory) no depletion of nutrients, which could have limited prokaryotic growth, was detectable, indicating that observed differences in outcomes are attributable to the test materials themselves. Other studies have previously highlighted that different plastic polymers can have differing biological effects, and the toxicity of PVC has been shown to be driven by the chemicals rather than the polymer itself.^55,56^ Since tire rubber and PVC are highly different regarding their chemical composition and physicochemical properties, differing effects on prokaryotes can also be expected.

The previous study further noticed that PE released a higher diversity of organic chemicals into the seawater compared to PVC pellets, though PVC leached significantly higher amounts (cumulative max. 88.2 μg per g PVC versus max. 0.74 μg per g PE). Organic target compounds in CMTT leachates exceeded PVC leachates by a factor of 10 (max. 858 μg per g CMTT), likely due in part to the differing particle sizes. Indeed, the average PVC pellet diameter (3.6 mm) is more comparable to the VCR and WCR particle size. Here, maximum cumulative concentrations of 240 μg per g VCR and 29.8 μg per g WCR were observed. Direct comparison of our results is, however, limited given certain differences in methodology (1 month leaching at 10 MPa vs 14 days leaching at 20 MPa in this study). Further, both studies employed targeted analysis instead of nontarget approaches, meaning that the observations are influenced by the choice of target chemicals.

The study using PVC and PE pellets generally observed a lower leaching of organic additives under high-hydrostatic pressure conditions. The authors specified, however, that this was only significant for higher molecular weight, nonpolar additives such as DEHP (diethylhexyl phthalate) and DiNP (diisononyl phthalate), not for lower molecular weight, more polar additives as DMP (dimethyl phthalate) and DEP (diethyl phthalate). In CMTT samples, high-hydrostatic pressure conditions lead to an increase of organic leachates compared to atmospheric pressure conditions (e.g., for *phenylguanidines* and *benzothiazoles*) or to no observed differences in leachate concentrations (e.g., for *phenylenediamines*). Thus, the summarized results of the previous and current study indicate that high-hydrostatic pressure might increase, decrease or have no effect on the leaching of organic compounds from polymer particles, depending on (i) the molecular weight of the chemicals, (ii) their water solubility, (iii) their degree of physical embedment in the polymer matrix and (iv) possibly other physicochemical properties of the chemicals and/or the polymers.

### Environmental Implications and Limitations of the Study

Although environmental concentrations of tire and road wear particles (TRWP) are typically much lower than those applied in this study (1 g/L), data on the occurrence of TRWP in deep-sea sediments is currently lacking, representing a major knowledge gap regarding the environmental fate of TRWP. Given that TRWPs are expected to sink and accumulate on the seafloor,^57^ it is plausible that local concentrations in deep-sea sediments may exceed those reported for surface waters. The selected concentration was further chosen to ensure consistency with a previous study using the same material, which focused on simulating photodegradation processes occurring in the surface layer of the marine environment.^20^ Applying a lower test material concentration would likely result in a lower total leachate concentration and altered leaching kinetics (e.g., due to partitioning effects and saturation effects at higher concentrations). The incorporation of a range of particle concentrations, in addition to the different particle sizes, should thus be considered for future studies.

To validate our findings and increase the environmental relevance, future studies would also benefit from using real TRWP instead of CMTT, given differences in particles size, density, organic content and amount of additive chemicals, among others. The smaller size of TRWP and thus their higher specific surface area could, for example, lead to a faster leaching of additive chemicals and thus to a quicker depletion of chemicals from the particles. On the other hand, the inclusion of crumb rubber as used in outdoor artificial sports fields represents a test material as it can be found in the environment. Further, the use of a test material preweathered under natural marine conditions (WCR) supports the environmental relevance of this study and allowed us to identify crumb rubber as a long-term leaching source of organic contaminants. Combined results of the seawater leaching experiment and the solvent extraction of WCR particles revealed that a substantial fraction of tire chemicals remains in the bulk material after 1 year seawater exposure, but becomes less accessible to water-driven desorption. If ingested by biota, this reservoir may become bioaccessible as gastrointestinal conditions (different pH, enzymes, surfactants) can mobilize compounds that are not water-accessible under seawater leaching tests.

Concentrations of leached chemicals were generally higher under biotic conditions, with opposite trends for chemicals prone to biotransformation processes. Similarly, the impact of the test material on the prokaryotic abundance indicates that these anthropogenic particles influence the very basis of marine ecosystems, including under deep-sea conditions. Until now, studies have mainly focused on the presence of tire particles and related chemicals in direct road runoff, surface waters or soils. Our results show, however, that if tire particles sink to the deep sea, the leaching of chemicals will continue and might even increase for certain compounds, depending on their physicochemical properties and their embedment or mobility within the polymer matrix, among others. By providing the first experimental evidence of pressure-driven differences in the release of chemicals from tire materials, this study increases current knowledge of how tire-derived pollutants behave across marine environments and provides a foundation for improved risk assessments and fate-and-transport modeling.

## Supplementary Material





## Notes

Supporting Information 1: Word document containing information on CMTT composition (Table S1) and particle size (Figure S1), spike-and-recovery test results (Figure S2), chemical details (Table S2), UPLC-HRMS instrumental method (Text S1), PO_4_^3–^ concentrations (Figure S3), SEM images (Figure S4), picture of CMTT agglomeration behavior (Figure S5), DOC concentrations (Table S3), solvent extraction of particles (Figure S6) (PDF)
Supporting Information 2: Excel file containing target chemical data from seawater leachates containing CMTT particles (Table S4), VCR or WCR particles (Table S5) and control samples (Table S6) (XLSX)

## Abbreviations

2-MBT: 2-mercaptobenzothiazole
2-MTBT: 2-(methylthio)­benzothiazole
2-(4-Mo)-BT: 2-(4-morpholinyl)­benzothiazole
2-OHBT: 2-hydroxybenzothiazole
4-HDPA: 4-hydroxydiphenylamine
6-PPD: *N*-(1,3-dimethylbutyl)-*N*′-phenyl-*p*-phenylenediamine
6-PPDQ: 6-PPD-quinone
BTSA: benzothiazole-2-sulfonic acid
CMTT: cryo-milled tire tread
CR: crumb rubber
DCH: dicyclohexylamine
DOC: dissolved organic carbon
DPG: 1,3-diphenylguanidine
DPPD: *N*,*N*-diphenyl-*p*-phenylendiamine
DW: deep-seawater
HMMM: hexa­(methoxymethyl)­melamine
HPBs: high-pressure bottles
MeDCH: *N*-methyldicyclohexylamine
NH2-BT: 2-aminobenzothiazole
NO–DPA: *N*-nitrosodiphenylamine
PE: polyethylene
PES: poly­(ether sulfone)
PG: phenylguanidine
PP: polypropylene
PTFE: polytetrafluoroethylene
PVC: polyvinyl chloride
SEM: scanning electron microscopy
SW: surface water
TP: tire particles
TPG: triphenylguanidine
TRWP: tire and road wear particles
TWP: tire wear particles
UPLC-MS: ultraperformance liquid chromatography–mass spectrometry
UPLC-TOF-MS: ultraperformance liquid chromatography time-of-flight mass-spectrometry
VCR: virgin crumb rubber
WCR: weathered crumb rubber.

## References

[1] Bergmann M., Wirzberger V., Krumpen T., Lorenz C., Primpke S., Tekman M. B., Gerdts G. (2017). High Quantities of Microplastic in
Arctic Deep-Sea Sediments from the HAUSGARTEN Observatory. Environ. Sci. Technol..

[2] Woodall L. C., Sánchez-Vidal A., Canals M., Paterson G. L. J., Coppock R., Sleight V., Calafat A., Rogers A. D., Narayanaswamy B. E., Thompson R. C. (2014). The Deep Sea Is a Major Sink for Microplastic Debris. R. Soc. Open Sci..

[3] Abel S. M., Primpke S., Int-Veen I., Brandt A., Gerdts G. (2021). Systematic
Identification of Microplastics in Abyssal and Hadal Sediments of
the Kuril–Kamchatka Trench. Environ.
Pollut..

[4] Katija K., Choy C. A., Sherlock R. E., Sherman A. D., Robison B. H. (2017). From the
Surface to the Seafloor: How Giant Larvaceans Transport Microplastics
into the Deep Sea. Sci. Adv..

[5] Justino A. K. S., Ferreira G. V. B., Eduardo L. N., Lenoble V., Fauvelle V., Schmidt N., Mincarone M. M., Sempéré R., Panagiotopoulos C., Frédou T., Lucena-Frédou F. (2022). The Role of Mesopelagic
Fishes as Microplastics Vectors across the Deep-Sea Layers from the
Southwestern Tropical Atlantic. Environ. Pollut..

[6] Ferreira G. V. B., Justino A. K. S., Eduardo L. N., Lenoble V., Fauvelle V., Schmidt N., Junior T. V., Frédou T., Lucena-Frédou F. (2022). Plastic in the Inferno: Microplastic Contamination
in Deep-Sea Cephalopods (*Vampyroteuthis infernalis* and *Abralia veranyi*) from the Southwestern Atlantic. Mar. Pollut. Bull..

[7] Krause S., Molari M., Gorb E. V., Gorb S. N., Kossel E., Haeckel M. (2020). Persistence of Plastic
Debris and Its Colonization
by Bacterial Communities after Two Decades on the Abyssal Seafloor. Sci. Rep..

[8] Hahladakis J. N., Velis C. A., Weber R., Iacovidou E., Purnell P. (2018). An Overview of Chemical Additives
Present in Plastics:
Migration, Release, Fate and Environmental Impact during Their Use,
Disposal and Recycling. J. Hazard. Mater..

[9] Hermabessiere L., Dehaut A., Paul-Pont I., Lacroix C., Jezequel R., Soudant P., Duflos G. (2017). Occurrence
and Effects of Plastic
Additives on Marine Environments and Organisms: A Review. Chemosphere.

[10] Coffin S., Dudley S., Taylor A., Wolf D., Wang J., Lee I., Schlenk D. (2018). Comparisons
of Analytical Chemistry and Biological
Activities of Extracts from North Pacific Gyre Plastics with UV-Treated
and Untreated Plastics Using in Vitro and in Vivo Models. Environ. Int..

[11] Paluselli A., Fauvelle V., Galgani F., Sempéré R. (2019). Phthalate
Release from Plastic Fragments and Degradation in Seawater. Environ. Sci. Technol..

[12] Sait S. T. L., Sørensen L., Kubowicz S., Vike-Jonas K., Gonzalez S. V., Asimakopoulos A. G., Booth A. M. (2021). Microplastic Fibres
from Synthetic Textiles: Environmental Degradation and Additive Chemical
Content. Environ. Pollut..

[13] Sørensen L., Groven A. S., Hovsbakken I. A., Del Puerto O., Krause D. F., Sarno A., Booth A. M. (2021). UV Degradation of
Natural and Synthetic Microfibers Causes Fragmentation and Release
of Polymer Degradation Products and Chemical Additives. Sci. Total Environ..

[14] Suhrhoff T. J., Scholz-Böttcher B. M. (2016). Qualitative
Impact of Salinity, UV
Radiation and Turbulence on Leaching of Organic Plastic Additives
from Four Common Plastics - A Lab Experiment. Mar. Pollut. Bull..

[15] Fauvelle V., Garel M., Tamburini C., Nerini D., Castro-Jiménez J., Schmidt N., Paluselli A., Fahs A., Papillon L., Booth A. M., Sempéré R. (2021). Organic Additive Release
from Plastic to Seawater Is Lower under Deep-Sea Conditions. Nat. Commun..

[16] Jan
Kole P., Löhr A. J., Van Belleghem F. G. A. J., Ragas A. M. J. (2017). Wear and Tear of Tyres: A Stealthy Source of Microplastics
in the Environment. Int J Environ Res Public
Health.

[17] Wang Y., Li X., Yang H., Wu Y., Pu Q., He W., Li X. (2024). A Review of Tire Wear Particles:
Occurrence, Adverse Effects, and
Control Strategies. Ecotoxicol. Environ. Saf..

[18] Weyrauch S., Seiwert B., Voll M., Wagner S., Reemtsma T. (2023). Accelerated
Aging of Tire and Road Wear Particles by Elevated Temperature, Artificial
Sunlight and Mechanical Stress — A Laboratory Study on Particle
Properties, Extractables and Leachables. Sci.
Total Environ..

[19] Hägg F., Herzke D., Nikiforov V. A., Booth A. M., Sperre K. H., Sørensen L., Creese M. E., Halsband C. (2023). Ingestion of Car Tire
Crumb Rubber and Uptake of Associated Chemicals by Lumpfish (*Cyclopterus lumpus*). Front. Environ.
Sci..

[20] Foscari A., Schmidt N., Seiwert B., Herzke D., Sempéré R., Reemtsma T. (2023). Leaching of
Chemicals and DOC from Tire Particles under
Simulated Marine Conditions. Front. Environ.
Sci..

[21] Zahn D., Mucha P., Zilles V., Touffet A., Gallard H., Knepper T. P., Frömel T. (2019). Identification
of Potentially Mobile
and Persistent Transformation Products of REACH-Registered Chemicals
and Their Occurrence in Surface Waters. Water
Res..

[22] Johannessen C., Helm P., Lashuk B., Yargeau V., Metcalfe C. D. (2022). The Tire
Wear Compounds 6PPD-Quinone and 1,3-Diphenylguanidine in an Urban
Watershed. Arch. Environ. Contam. Toxicol..

[23] Chibwe L., Parrott J. L., Shires K., Khan H., Clarence S., Lavalle C., Sullivan C., O’Brien A. M., De Silva A. O., Muir D. C. G., Rochman C. M. (2022). A Deep
Dive into
the Complex Chemical Mixture and Toxicity of Tire Wear Particle Leachate
in Fathead Minnow. Environ. Toxicol. Chem..

[24] Tian Z., Zhao H., Peter K. T., Gonzalez M., Wetzel J., Wu C., Hu X., Prat J., Mudrock E., Hettinger R., Cortina A. E., Biswas R. G., Kock F. V. C., Soong R., Jenne A., Du B., Hou F., He H., Lundeen R., Gilbreath A., Sutton R., Scholz N. L., Davis J. W., Dodd M. C., Simpson A., McIntyre J. K., Kolodziej E. P. (2021). A Ubiquitous Tire Rubber-Derived Chemical Induces Acute
Mortality in Coho Salmon. Science.

[25] Lane R. F., Smalling K. L., Bradley P. M., Greer J. B., Gordon S. E., Hansen J. D., Kolpin D. W., Spanjer A. R., Masoner J. R. (2024). Tire-Derived
Contaminants 6PPD and 6PPD-Q: Analysis, Sample Handling, and Reconnaissance
of United States Stream Exposures. Chemosphere.

[26] Maurer L., Carmona E., Machate O., Schulze T., Krauss M., Brack W. (2023). Contamination Pattern
and Risk Assessment of Polar Compounds in Snow
Melt: An Integrative Proxy of Road Runoffs. Environ. Sci. Technol..

[27] Wagner S., Funk C. W., Müller K., Raithel D. J. (2024). The Chemical Composition
and Sources of Road Dust, and of Tire and Road Wear Particles–A
Review. Sci. Total Environ..

[28] Wik A., Dave G. (2009). Occurrence
and Effects of Tire Wear Particles in the Environment
- A Critical Review and an Initial Risk Assessment. Environ. Pollut..

[29] Goßmann I., Halbach M., Scholz-Böttcher B. M. (2021). Car and
Truck Tire
Wear Particles in Complex Environmental Samples – A Quantitative
Comparison with “Traditional” Microplastic Polymer Mass
Loads. Sci. Total Environ..

[30] Rausch J., Jaramillo-Vogel D., Perseguers S., Schnidrig N., Grobéty B., Yajan P. (2022). Automated Identification and Quantification
of Tire Wear Particles (TWP) in Airborne Dust: SEM/EDX Single Particle
Analysis Coupled to a Machine Learning Classifier. Sci. Total Environ..

[31] Zeng L., Li Y., Sun Y., Liu L.-Y., Shen M., Du B. (2023). Widespread
Occurrence and Transport of p-Phenylenediamines and Their Quinones
in Sediments across Urban Rivers, Estuaries, Coasts, and Deep-Sea
Regions. Environ. Sci. Technol..

[32] Halle L. L., Palmqvist A., Kampmann K., Jensen A., Hansen T., Khan F. R. (2021). Tire Wear Particle and Leachate Exposures from a Pristine
and Road-Worn Tire to *Hyalella azteca*: Comparison
of Chemical Content and Biological Effects. Aquat. Toxicol..

[33] Halsband C., Sørensen L., Booth A. M., Herzke D. (2020). Car Tire Crumb Rubber:
Does Leaching Produce a Toxic Chemical Cocktail in Coastal Marine
Systems?. Front. Environ. Sci..

[34] McIntyre J. K., Prat J., Cameron J., Wetzel J., Mudrock E., Peter K. T., Tian Z., Mackenzie C., Lundin J., Stark J. D., King K., Davis J. W., Kolodziej E. P., Scholz N. L. (2021). Treading Water: Tire Wear Particle
Leachate Recreates an Urban Runoff Mortality Syndrome in Coho but
Not Chum Salmon. Environ. Sci. Technol..

[35] Müller K., Hübner D., Huppertsberg S., Knepper T. P., Zahn D. (2022). Probing the
Chemical Complexity of Tires: Identification of Potential Tire-Borne
Water Contaminants with High-Resolution Mass Spectrometry. Sci. Total Environ..

[36] Rauert C., Charlton N., Okoffo E. D., Stanton R. S., Agua A. R., Pirrung M. C., Thomas K. V. (2022). Concentrations
of Tire Additive Chemicals
and Tire Road Wear Particles in an Australian Urban Tributary. Environ. Sci. Technol..

[37] Seiwert B., Nihemaiti M., Troussier M., Weyrauch S., Reemtsma T. (2022). Abiotic Oxidative
Transformation of 6-PPD and 6-PPD Quinone from Tires and Occurrence
of Their Products in Snow from Urban Roads and in Municipal Wastewater. Water Res..

[38] Thomas J., Moosavian S. K., Cutright T., Pugh C., Soucek M. D. (2022). Investigation
of Abiotic Degradation of Tire Cryogrinds. Polym.
Degrad. Stab..

[39] Fohet L., Andanson J. M., Charbouillot T., Malosse L., Leremboure M., Delor-Jestin F., Verney V. (2023). Time-Concentration Profiles of Tire
Particle Additives and Transformation Products under Natural and Artificial
Aging. Sci. Total Environ..

[40] Lefevre, D. EMSO LIGURE OUEST 2022 - PP Cruise, RV Pourquoi Pas ? 2022 10.17600/18001344.

[41] Aminot Y., Fuster L., Pardon P., Le Menach K., Budzinski H. (2018). Suspended Solids Moderate the Degradation and Sorption
of Waste Water-Derived Pharmaceuticals in Estuarine Waters. Sci. Total Environ..

[42] Garel M., Bonin P., Martini S., Guasco S., Roumagnac M., Bhairy N., Armougom F., Tamburini C. (2019). Pressure-Retaining
Sampler and High-Pressure Systems to Study Deep-Sea Microbes under
in Situ Conditions. Front. Microbiol..

[43] Roumagnac M., Pradel N., Bartoli M., Garel M., Jones A. A., Armougom F., Fenouil R., Tamburini C., Ollivier B., Summers Z. M., Dolla A. (2020). Responses to the Hydrostatic
Pressure of Surface and Subsurface Strains of *Pseudothermotoga
elfii* Revealing the Piezophilic Nature of the Strain Originating
from an Oil-Producing Well. Front. Microbiol..

[44] Klöckner P., Seiwert B., Weyrauch S., Escher B. I., Reemtsma T., Wagner S. (2021). Comprehensive Characterization of
Tire and Road Wear
Particles in Highway Tunnel Road Dust by Use of Size and Density Fractionation. Chemosphere.

[45] Marie D., Rigaut-Jalabert F., Vaulot D. (2014). An Improved Protocol for Flow Cytometry
Analysis of Phytoplankton Cultures and Natural Samples. Cytometry Part A.

[46] Marie, D. ; Simon, N. ; Guillou, L. ; Partensky, F. ; Vaulot, D. . In Living Color: Protocols in Flow Cytometry and Cell Sorting; Diamond, R. A. , DeMaggio, S. , Eds.; Springer, 2000.

[47] Sempéré R., Para J., Tedetti M., Charrière B., Mallet M. (2015). Variability of Solar Radiation and CDOM in Surface
Coastal Waters of the Northwestern Mediterranean Sea. Photochem. Photobiol..

[48] Nagata T., Tamburini C., Arístegui J., Baltar F., Bochdansky A. B., Fonda-Umani S., Fukuda H., Gogou A., Hansell D. A., Hansman R. L., Herndl G. J., Panagiotopoulos C., Reinthaler T., Sohrin R., Verdugo P., Yamada N., Yamashita Y., Yokokawa T., Bartlett D. H. (2010). Emerging Concepts
on Microbial Processes in the Bathypelagic Ocean - Ecology, Biogeochemistry,
and Genomics. Deep Sea Res., Part II.

[49] Dittmar S., Weyrauch S., Reemtsma T., Eisentraut P., Altmann K., Ruhl A. S., Jekel M. (2025). Settling Velocities
of Tire and Road Wear Particles: Analyzing Finely Graded Density Fractions
of Samples from a Road Simulator and a Highway Tunnel. Environ. Sci. Technol..

[50] Santinelli C., Sempéré R., Van Wambeke F., Charrière B., Seritti A. (2012). Organic Carbon Dynamics
in the Mediterranean
Sea: An Integrated Study. Global Biogeochem.
Cycles.

[51] Ignatz-Hoover F., To B. H., Datta R. N., De Hoog A. J., Huntink N. M., Talma A. G. (2003). Chemical Additives
Migration in Rubber. Rubber Chem. Technol..

[52] Reemtsma T., Fiehn O., Kalnowski G., Jekel M. (1995). Microbial Transformations
and Biological Effects of Fungicide-Derived Benzothiazoles Determined
in Industrial Wastewater. Environ. Sci. Technol..

[53] Foscari A., Seiwert B., Zahn D., Schmidt M., Reemtsma T. (2024). Leaching of
Tire Particles and Simultaneous Biodegradation of Leachables. Water Res..

[54] Xu Q., Li G., Fang L., Sun Q., Han R., Zhu Z., Zhu Y. G. (2023). Enhanced Formation
of 6PPD-Q during the Aging of Tire
Wear Particles in Anaerobic Flooded Soils: The Role of Iron Reduction
and Environmentally Persistent Free Radicals. Environ. Sci. Technol..

[55] Zimmermann L., Göttlich S., Oehlmann J., Wagner M., Völker C. (2020). What Are the
Drivers of Microplastic Toxicity? Comparing the Toxicity of Plastic
Chemicals and Particles to *Daphnia magna*. Environ. Pollut..

[56] Zimmermann L., Dierkes G., Ternes T. A., Völker C., Wagner M. (2019). Benchmarking the in vitro Toxicity
and Chemical Composition
of Plastic Consumer Products. Environ. Sci.
Technol..

[57] Mattsson K., de Lima J. A., Wilkinson T., Järlskog I., Ekstrand E., Sköld Y. A., Gustafsson M., Hassellöv M. (2023). Tyre and Road Wear Particles from
Source to Sea. Microplast. Nanoplast..

