# Supplementary material for: Aging of Tire Particles in Deep-Sea Conditions: Interactions between Hydrostatic Pressure, Prokaryotic Growth and Chemical Leaching
Source: Environ Sci Technol. 2025 Sep 17;59(38):20748–60. doi: 10.1021/acs.est.5c10705 (PMC12490020; doi:10.1021/acs.est.5c10705)
Supplement: Supplementary file 1 [file es5c10705_si_001.pdf]

Supporting Information 1

Aging of tire particles in deep-sea conditions: interactions between hydrostatic pressure, prokaryotic growth and chemical leaching

Natascha Schmidt[1,2], Aurelio Foscari[3], Dorte Herzke[1], Marc Garel[2], Christian Tamburini[2], Bettina Seiwert[3], Thorsten Reemtsma[3,4], Richard Sempéré[5,2]

[1] NILU, The FRAM Centre, P.O. Box 6606, Tromsø, 9296, Norway

[2] Aix Marseille Univ, Université de Toulon, CNRS, IRD, MIO, Marseille, 13009, France

[3] Helmholtz Centre for Environmental Research—UFZ, Department of Environmental Analytical Chemistry, Leipzig, 04318, Germany

[4] Institute for Analytical Chemistry, University of Leipzig, Leipzig, 04109, Germany

[5] Aix-Marseille Univ., CNRS, LCE, UM 7376, Ocean Sciences Institute, Marseille, 13003, France

Contents (pages S2-S11):

Table S1. List of tire types and brands used for the cryo-milled tire tread mixture.

Figure S1: (A) The cryo-milled tire tread (CMTT) particle size range in number (left) and volume (right) distribution, and (B) the SEM images at 250X (left) and 1000X (right) magnification.

Figure S2. Recoveries (n = 3) obtained from the filter test (PES, 0.22 µm pore size) using a standard mixture. For comparison, the same procedure was performed without the filter to evaluate potential matrix effects. Both tests were conducted under identical conditions, including the same filtration system, sample volume (130 mL), and seawater medium.

Table S2. Selected organic compounds analyzed by LC-MS

Text S1: Analysis by UPLC-HRMS – instrumental method

Figure S3: PO_4^{3-} concentrations (in µM) in surface waters (SW, upper panel) and deep-sea waters (DW, lower panel) samples under biotic (right) and abiotic (left) conditions. Due to a restricted amount of test material available for the experiment, abiotic WCR samples t_1-t_3 (6 h, 24 h, 7 d) are lacking.

Figure S4. Scanning electron microscope (SEM) images showing the presence of prokaryotes and other organic materials on the surface of cryo-milled tire tread (CMTT), virgin and weathered crumb rubber (VCR and WCR) particles after the experimental time (14 days) aged in both abiotic and biotic conditions.

Figure S5: Behaviour of CMTT in surface water samples kept at atmospheric pressure conditions (A) and in deep-sea water samples kept at 20 MPa (B).

Table S3. Dissolved organic carbon (DOC) concentrations in µM during time course experiment.

Figure S6. Graphs showing the total amount of compounds measured after solvent extraction (methanol) of the aged material in both abiotic and biotic conditions. The quantified compounds are grouped into their belonging chemical class (*phenylguanidines, benzothiazoles, phenylendiamines and other amines*).

The following information is provided on different sheets of a separate Excel file denoted as Supporting Information 2:

Table S4. Concentrations of the quantified compounds listed in Table S2 during the exposure experiment in the cryo-milled tire tread (CMTT) samples.
Please see Excel-table "Tables S4_CMTT"

Table S5. Concentrations of the quantified compounds listed in Table S2 during the exposure experiment in the virgin and weathered crumb rubber (VCR and WCR, repectively).

Please see Excel-table "Tables S5_VCR&WCR"

Table S6. Concentrations of the quantified compounds listed in Table S2 during the exposure experiment in the control (CTR) samples.
Please see Excel-table "Tables S6_CTR"

Table S1: List of tire types and brands used for the cryo-milled tire tread mixture.

Sample	Season	Details
TP1	all-season	Falken outside Euroall Season 225/50 R17 98 V
TP2	winter	WINTEC PN150 165/65 R15 91T M+S
TP3	winter	Goodyear Vector 5+ M+S 185/65 R15 88T
TP4	winter	Fulda Kristall Montero 2 M+S 195/60 R15 88T
TP5	winter	Continental ContiWinter Contact TS830 205/55 R16
TP6	summer	tire rubber summer (typical mixture)
TP7	winter	tire rubber winter (typical mixture)
TP8	not specified	Continental Germany
TP9	not specified	Triangle, China
TP10	not specified	Wanlitire, China
TP11	not specified	Cheng Shin, China
TP12	summer	Bridgestone DriveGuard 225/40R18 92Y DRGSFZ 67854 VRT7
TP13	winter	Pirelli Sottozero 3 225/40 R18 92Y M+S extra load studless tubeless
TP14	winter	Fulda Kristall Montero 3 205/65 R15 94T M+S
TP15	all-season	Continental VancoFourSeason 2 235/65 R16 C
TP16	summer	Dunlop SP Sport Maxx GT 235/65 R17
TP17	summer	Sava intensa uhp 225/50 R16 92W
TP18	summer	Continental ContiSportContact 5 235/45 R17 94W
TP19	summer	Hankook VentusPrime 3 205/55R16 91V
TP20	summer	Semperit Speed-Life 195/50 R15 82H alpine proven

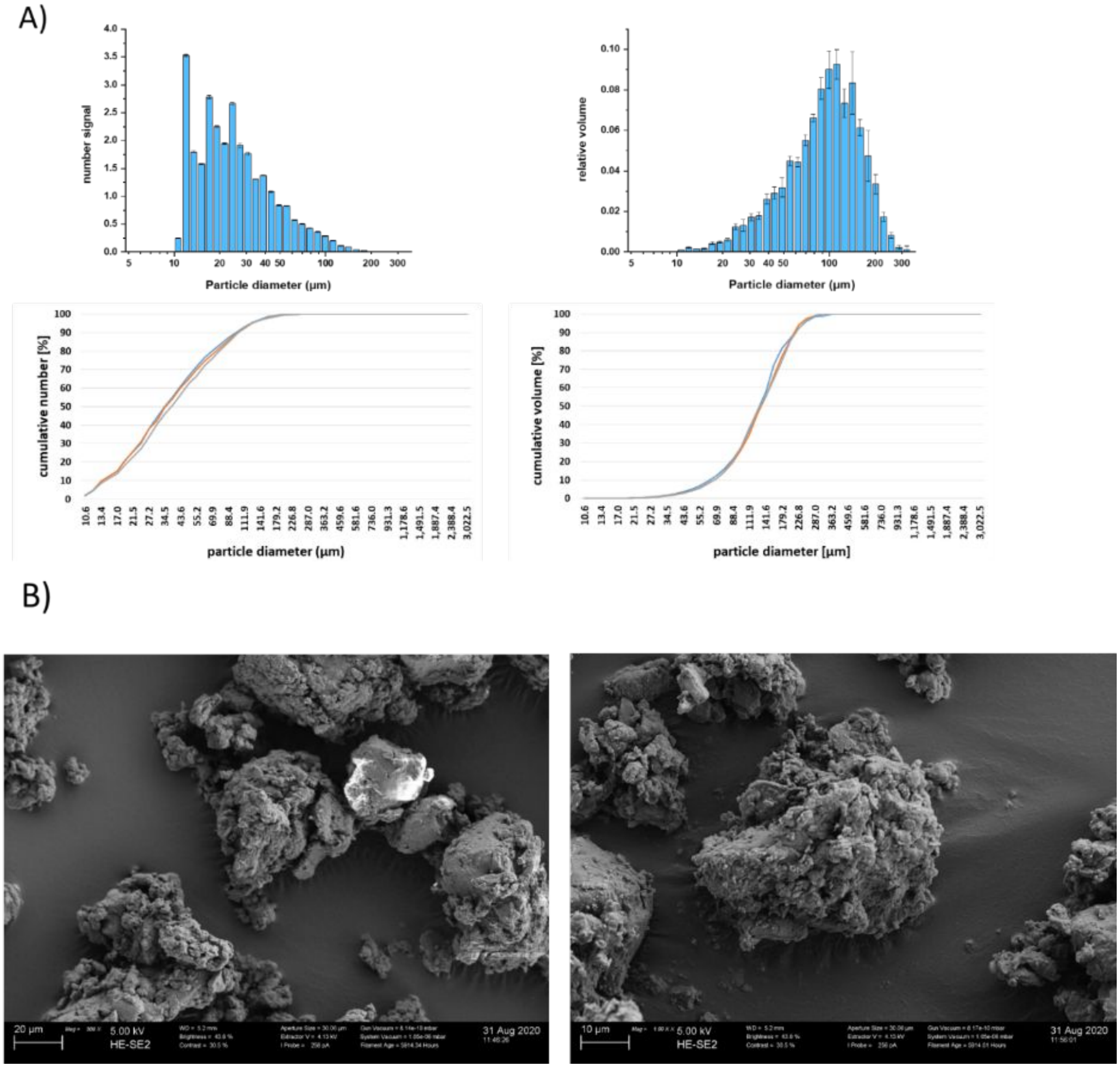

Figure S1: (A) The cryo-milled tire tread (CMTT) particle size range in number (left) and volume (right) distribution, and (B) the SEM images at 250X (left) and 1000X (right) magnification.

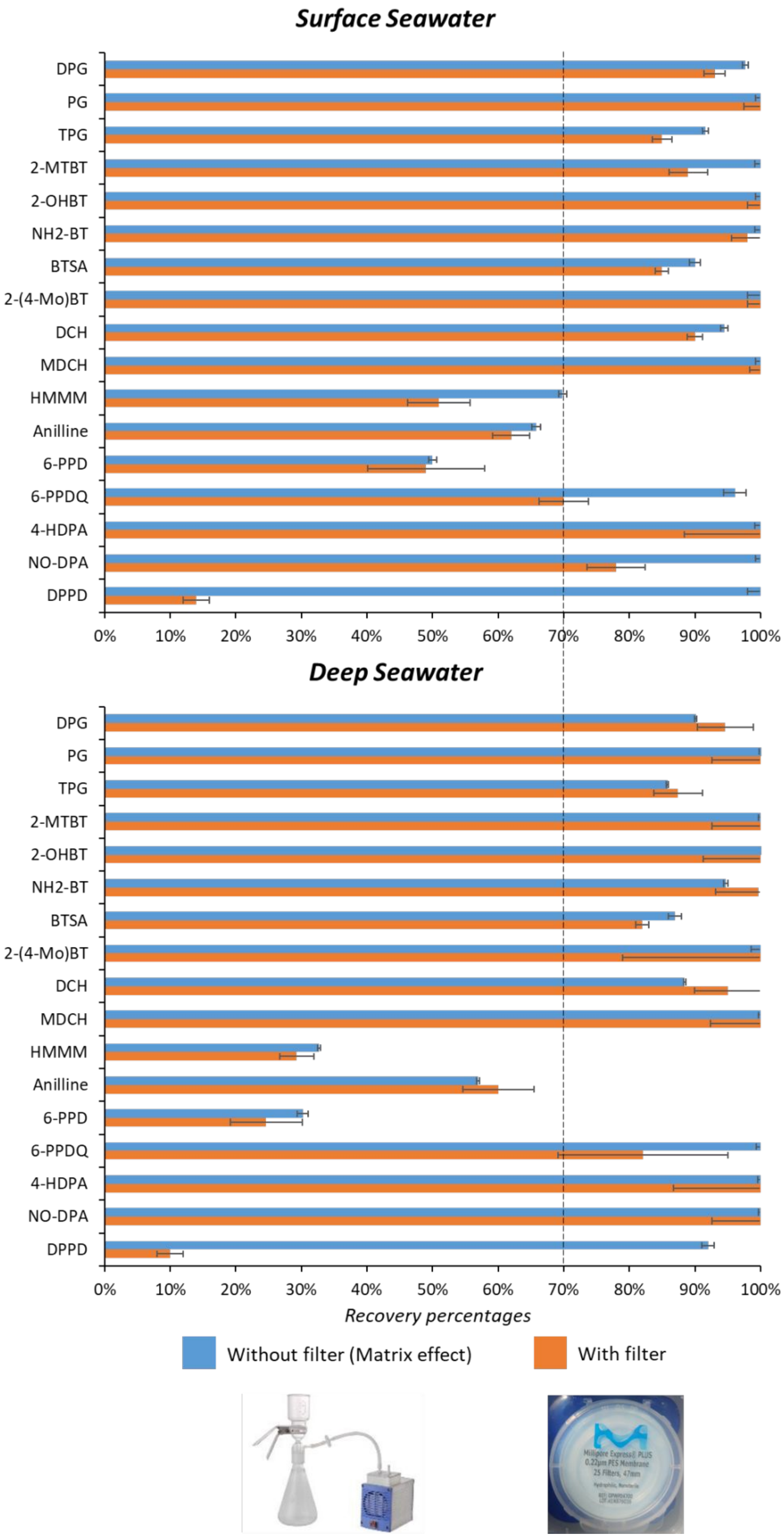

Figure S2. Recoveries (n = 3) obtained from the filter test (PES, 0.22 µm pore size) using a standard mixture. For comparison, the same procedure was performed without the filter to evaluate potential matrix effects. Both tests were conducted under identical conditions, including the same filtration system, sample volume (130 mL), and seawater medium.

Table S2. Selected organic compounds analyzed by LC-MS

Abbreviation	CAS	Name	Sum Formula	RT [min]	Exact mass (*m/z*, $[M+H]^+$ or M-H]-)	standard for quantification	LOQ ng/ml	Supplier	Purity grade
DPG	102-06-7	Diphenylguanidine	$C_{13}H_{13}N_3$	4.83	212.119	yes	0.1	Merck (Darmstadt, germany)	97%
PG	2002-16-6	Phenylguanidine	$C_7H_9N_3$	2.62	136.087	yes	0.03	BLD pharm	97%
Aniline	62-53-3	Benzenamine	$C_6H_5NH_2$	1.34	94.065	yes	0.3	Sigma-aldrich (Schnelldorf, Germany)	97%
2-MTBT	615-22-5	2-(Methylthio)benzothiazole	$C_8H_7NS_2$	9.52	182.01	yes	0.3	abcr GmbH (Karlsruhe, Germany)	98%
NH_2-BT	136-95-8	2-Aminobenzothiazole	$C_7H_6N_2S$	3.82	151.033	yes	0.03	Sigma-aldrich (Schnelldorf, Germany)	97%
2-(4-Mo)-BT	4225-26-7	2-(4-morpholinyl)benzothiazole	$C_{11}H_{12}N_2OS$	8.39	221.075	yes	1	abcr GmbH (Karlsruhe, Germany)	95%
2-OHBT	934-34-9	2-Hydroxybenzothiazole	C_7H_5NOS	6.88	152.017	yes	0.03	Sigma-aldrich (Schnelldorf, Germany)	98%
BTSA	941-57-1	Benzothiazole-2-sulfonic acid	$C_7H_5NO_3S_2$	4.96	213.9633 (-)	yes	0.1	Sigma-aldrich (Schnelldorf, Germany)	98%
6-PPD	793-24-8	N-(1,3-dimethylbutyl)-N′-phenyl-1,4-phenylenediamine	$C_{18}H_{24}N_2$	8.39	269.202	yes	0.3	abcr GmbH (Karlsruhe, Germany)	98%
4-HDPA	122-37-2	4-Hydroxydiphenylamine	$C_{12}H_{11}NO$	7.85	186.0919	yes	0.03	Alfa Aesar (Kandel, Germany)	98%
6-PPD-Q	8026-48-0	6-PPD-quinone	$C_{18}H_{22}N_2O_2$	10.96	299.176	yes	0.02	HPC Standards GmbH (Borsforf, Germany)	99%
DPPD	74-31-7	N,N′-Diphenyl-p-phenylenediamine	$C_{18}H_{16}N_2$	10.72	260.1313	yes	0.1	Sigma-aldrich (Schnelldorf, Germany)	98%
TPG	101-01-9	Triphenylguanidine	$C_{19}H_{17}N_3$	7.25	288.149	yes	0.1	J&K Scientific	98%
NO-DPA	86-30-6	Nitroso-diphenylamine	$C_{12}H_{10}N_2O$	8.8	199.089	yes	0.1	abcr GmbH (Karlsruhe, Germany)	95%
MDCH	7560-83-0	Methyldicyclohexylamine	$C_{13}H_{25}N$	5.72	196.2061	yes	0.03	Sigma-aldrich (Schnelldorf, Germany)	98%
HMMM	3089-11-0	Hexamethoxymethylmelamine	$C_{15}H_{30}N_6O_6$	8.63	413.2125	yes	0.03	abcr GmbH (Karlsruhe, Germany)	98%
DCH	101-83-7	Dicyclohexylamine	$C_{12}H_{23}N$	5.79	182.1909	no	0.1	Sigma-aldrich (Schnelldorf, Germany)	99%

Text S1: Analysis by UPLC-HRMS – instrumental method

For the reversed-phase analysis, an ACQUITY ultra performance liquid chromatography (UPLC) connected to a Xevo G2-XS quadrupole time-of-flight (QTof) mass spectrometry (Waters, Eschborn, Germany) was used. The injection volume was 10 µL. The UPLC separation was achieved using an ACQUITY UPLC HSS T3 column (100 × 2.1, 1.7 µm) at a flow rate of 0.45 mL min^{-1}. The column temperature was set to 45 °C. The mobile phase consisted of (A) water (0.1% formic acid) and (B) methanol (0.1% formic acid). The following gradient was applied: 0–0.25 min, 2% B; 12.25–15 min, 99% B; 15.1–17 min, 2% B. Samples were analysed using above instruments in positive and negative electrospray ionization modes (separate runs) following the same HRMS parameters. A lock-spray containing leucine enkephalin was continuously infused during measurement. The source settings include capillary voltage of 0.7 kV in positive and -2 kV in negative ionisation modes, source temperature at 140 °C, and desolvation temperature at 550 °C. The sampling cone voltage and source offset were set as 20 V and 50 V, respectively. Nitrogen and argon were used as cone and collision gases, respectively. The desolvation gas flow was 950 L h^{-1}. The data was recorded in sensitivity mode (resolution approx. 20000) as centroid data with a 0.15 s scan time over the mass range m/z 50 to m/z 1200. The MSE acquisition was performed to simultaneously collect two data sets: a low-collision-energy scan (4 eV) to obtain parent ion information and an elevated-collision-energy scan (15–35 eV) to get all fragment ions.

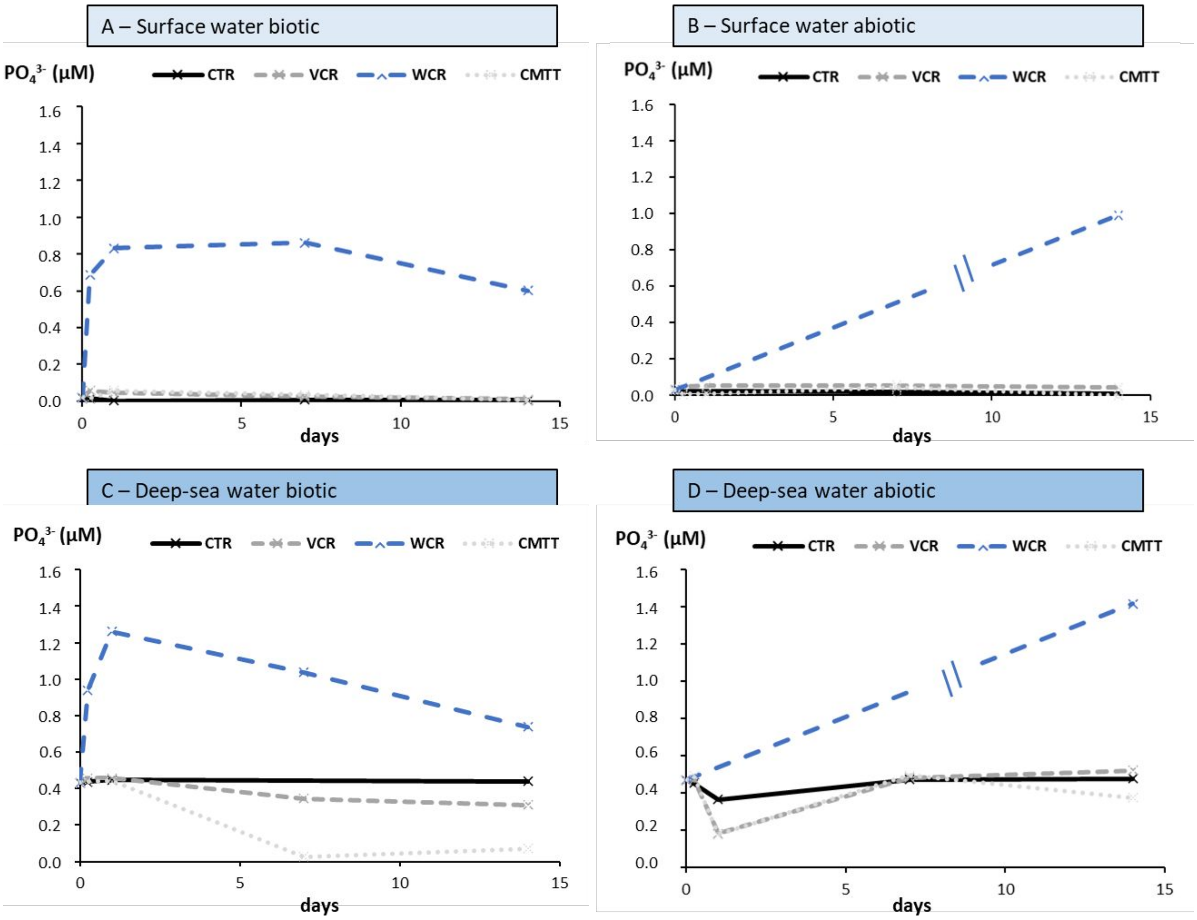

Figure S3: PO_4^{3-} concentrations (in µM) in SW (upper panel) and DW (lower panel) samples under biotic (right) and abiotic (left) conditions. Due to a restricted amount of test material available for the experiment, abiotic WCR samples t_1-t_3 (6 h, 24 h, 7 d) are lacking.

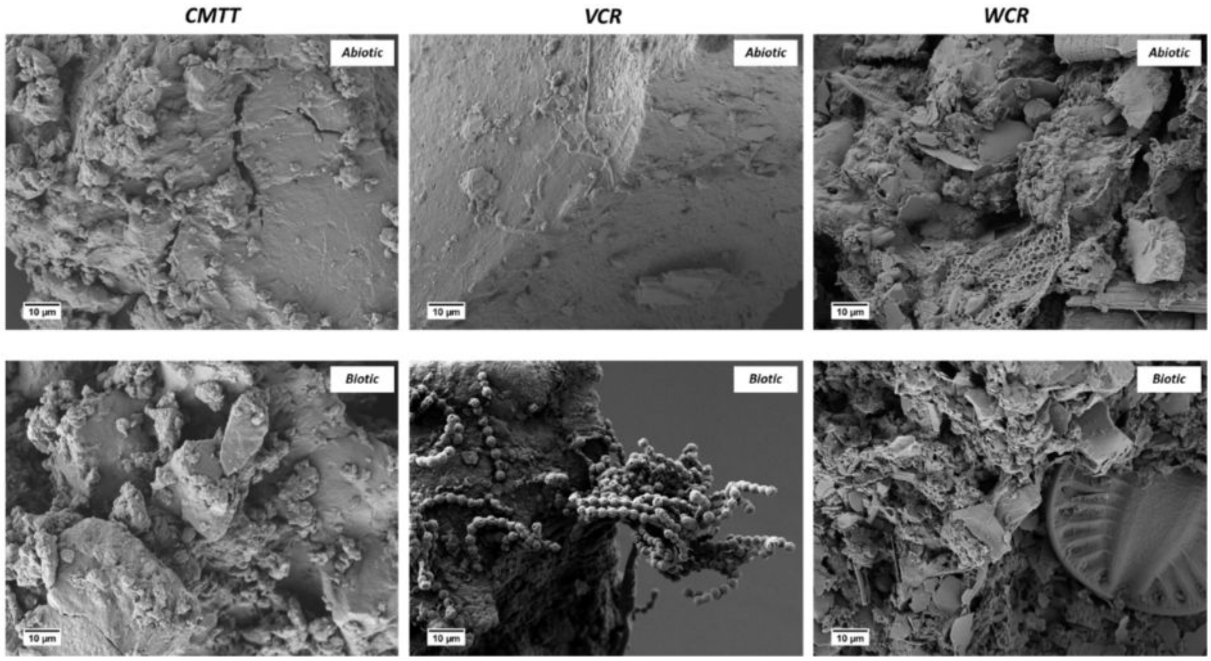

Figure S4. Scanning electron microscope (SEM) images showing the presence of bacteria and other organic materials on the surface of cryo-milled tire tread (CMTT), virgin and weathered crumb rubber (VCR and WCR) particles after the experimental time (14 days) aged in both abiotic and biotic conditions. On the WCR surface, the presence of a residual biofilm can be noticed.

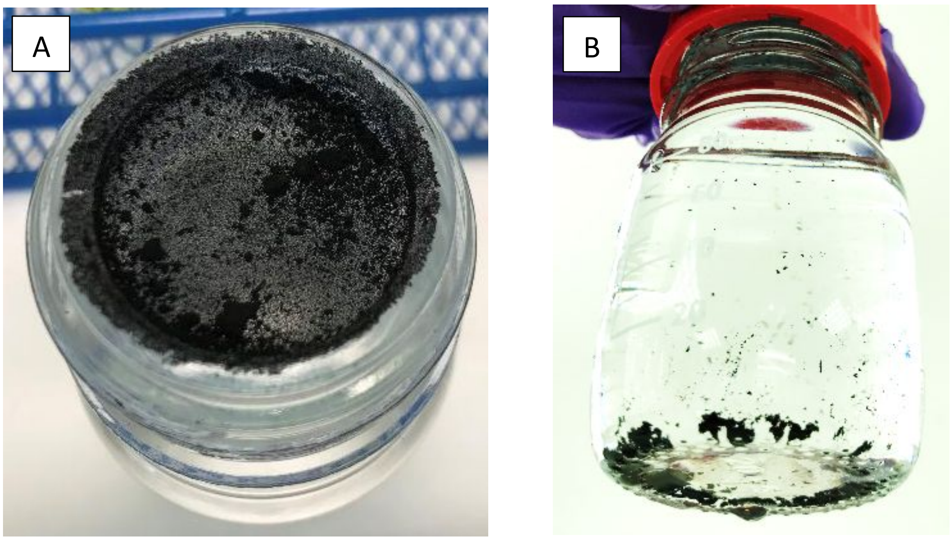

Figure S5: Behaviour of CMTT in surface water samples kept at atmospheric pressure conditions (A) and in deep-sea water samples kept at 20 MPa (B).

Table S3: Dissolved organic carbon (DOC) concentrations in µM during time course experiment. Empty cells indicate that sampling points were not made due to limitations concerning availability of test material (WCR). "CTR" represents control seawater samples without any test material.

	**t_0** **0 h**	**t_1** **6 h**	**t_2** **24 h**	**t_3** **7 d**	**t_4** **14 d**
SW biotic					
CTR	59	50	48	49	50
CMTT	-	80	101	148	159
VCR	-	75	97	94	89
WCR	-	78	78	72	74
DW biotic					
CTR	44	35	36	36	37
CMTT	-	116	137	161	168
VCR	-	63	61	65	65
WCR	-	57	68	61	54
SW abiotic					
CTR	49	47	52	56	55
CMTT	-	80	101	139	141
VCR	-	68	83	94	95
WCR	-				105
DW abiotic					
CTR	44	45	46	46	45
CMTT	-	112	124	175	185
VCR	-	64	72	83	83
WCR	-				112

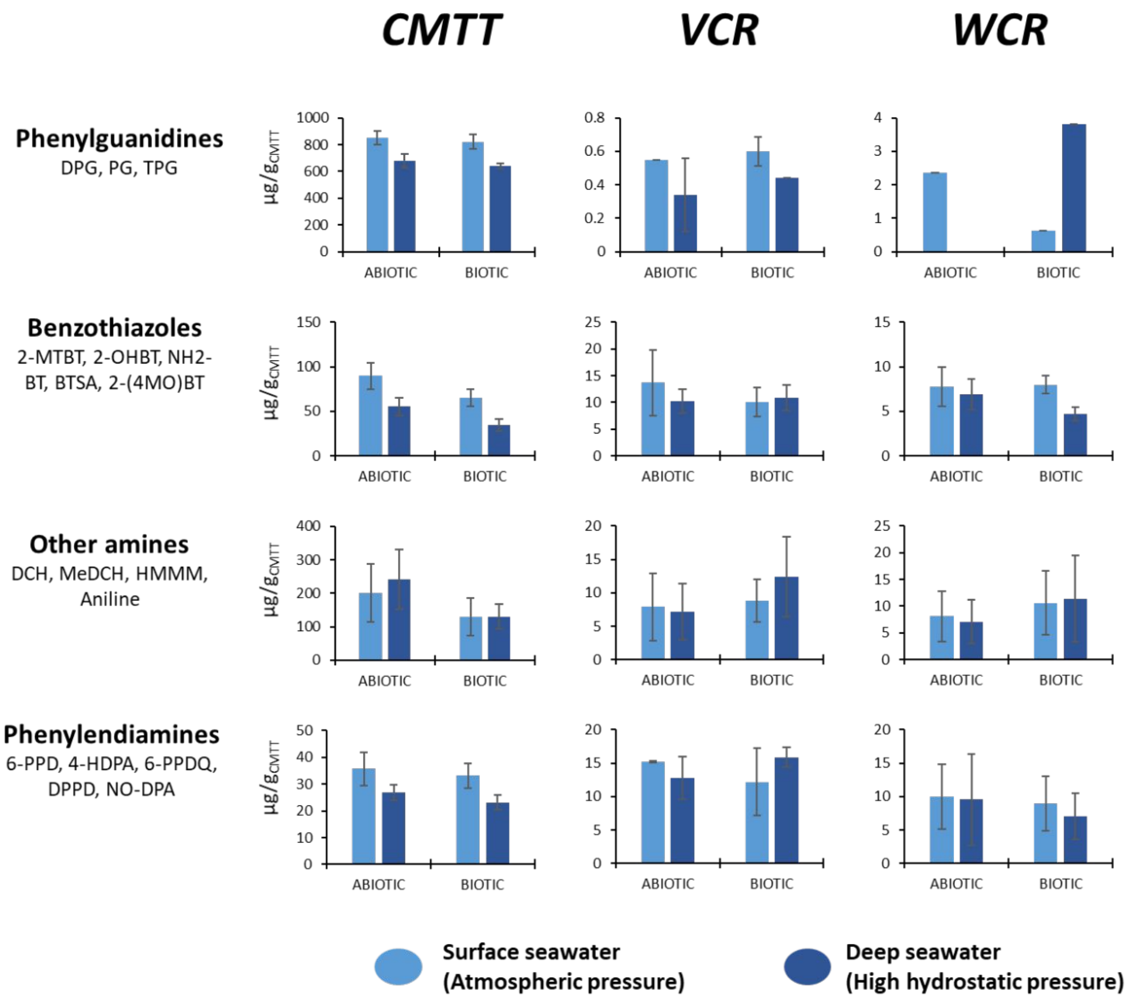

Figure S6. Graphs showing the total amount of compounds measured after solvent extraction (methanol) of the aged material in both abiotic and biotic conditions. The quantified compounds are grouped into their belonging chemical class (*phenylguanidines, benzothiazoles, phenylendiamines and other amines*).
